# Aquatic Thermal and Photochemical Reactivity of *N*‑(1,3-Dimethylbutyl)‑*N*′‑phenyl‑*p*‑phenylenediamine (6PPD), *N*‑Isopropyl‑*N*′‑phenyl‑*p*‑phenylenediamine (IPPD), and 6PPD-quinone

**DOI:** 10.1021/acs.est.4c12896

**Published:** 2025-06-16

**Authors:** Kathryn L. Platt, Oleksandr Yushchenko, Juliana R. Laszakovits, Yiwen Zhang, Nicholas C. Pflug, Kristopher McNeill

**Affiliations:** † Institute of Biogeochemistry and Pollutant Dynamics, 27219 ETH Zurich, 8092 Zurich, Switzerland; ‡ Department of Chemistry, SUNY ESF, Syracuse, New York 13210, United States

**Keywords:** tire-derived pollutants, photochemical quantum yields, triplet-state organic matter, dark oxidation

## Abstract

A ubiquitously used tire rubber antidegradant, 6PPD (*N*-(1,3-dimethylbutyl)-*N*′-phenyl-*p*-phenylenediamine), and its toxic ozonation product, 6PPD-quinone (*N*-(1,3-dimethylbutyl)-*N*′-phenyl-*p*-phenylenediamine quinone), have become recognized as important environmental pollutants since 6PPD-quinone (6PPD-Q) was identified as the likely cause of decades of mass Coho salmon kills. The reactivity of 6PPD, 6PPD-Q, and similar phenylenediamines requires study to better understand their environmental fate. This study explores the aquatic reactivity of 6PPD, *N*-isopropyl-*N*′-phenyl-1,4-phenylenediamine (IPPD), and 6PPD-Q through thermal and photochemical pathways using both steady-state photochemistry and time-resolved laser spectroscopy techniques. 6PPD was found to rapidly degrade in the dark, with its degradation rate being highly dependent on the pH, temperature, and oxygen concentrations. IPPD behaves similarly to 6PPD. In contrast, 6PPD-Q is much more stable in the dark. All three chemicals are degraded via direct photochemistry. Regarding indirect photochemistry, ^3^CDOM* plays a role in the degradation of 6PPD and IPPD but not 6PPD-Q, while ^1^O_2_ does not play a significant role for any of the compounds. Reaction rate constants are reported as well as 6PPD-Q molar yields from 6PPD, which were minimal for all aqueous pathways examined. 6PPD-Q may have a longer environmental lifetime as there are fewer degradation pathways. This research will help us to better understand and control these chemicals in the environment.

## Graphical abstract

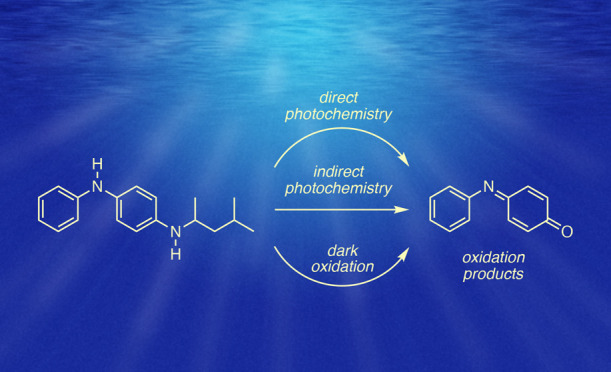

## 1 Introduction

The toxic chemical 6PPD-quinone (*N*-(1,3-dimethylbutyl)-*N*′-phenyl-*p*-phenylenediamine quinone) was first discovered in 2020 and identified as the likely primary toxin causing urban watershed toxic syndrome in Coho salmon ().^1^ 6PPD-quinone (6PPD-Q) forms through the reaction of 6PPD (*N*-(1,3-dimethylbutyl)-*N*′-phenyl-*p*-phenylenediamine) with ozone.^1^ 6PPD is an antidegradant added to tires and other rubber products to prolong their lifespan. Since discovering the harm that 6PPD and 6PPD-Q can cause, a surge of related research has emerged.^2–4^ A large portion of the work explores the toxicity of these compounds to other fish and aquatic organisms. Another portion of the research is devoted to identifying environmental concentrations of 6PPD and 6PPD-Q, along with other paraphenylenediamines (PPDs) and their corresponding PPD-quinones. The compounds have been found in nearly every environmental compartment, including water, air, soil, sediment, dust, surface runoff, and wastewater treatment plant effluent,^5–13^ as well as human urine.^14^ The geographic spread of these studies is extensive, including the USA, Canada, Germany, Australia, China, and more. The effects and ubiquity of these chemicals in our environment are firmly established, and therefore, understanding their reactivity and fate is of key importance.

The aquatic thermal and photochemical reactivity of these chemicals is one control of their environmental persistence. PPDs are prone to (nonphotochemical) oxidation in air due to their low oxidation potential.^15^ The dark degradation of 6PPD has been previously explored.^13,16,17^ However, the efficiency of this process can be influenced by the temperature and oxygen concentrations, which can vary greatly based on location and season, and this is not well-characterized.

The photochemical degradation of environmental pollutants can be classified into direct and indirect photochemistry. Direct photochemistry occurs when the pollutant itself absorbs light and undergoes a reaction.^18^ Indirect photochemistry occurs through absorption of light by other molecules in the water, which results in photochemically produced reactive intermediates (PPRI) that react with pollutants to cause degradation.^19^ In the aquatic environment, chromophoric dissolved organic matter (CDOM) absorbs sunlight to form reactive species such as excited triplet states of CDOM (^3^CDOM*) and singlet oxygen (^1^O_2_).^20^ The recent interest in the fate of these chemicals has led to a few studies that have investigated aspects of the photochemical degradation of 6PPD^13,16^ and 6PPD-Q.^21,22^

A central question regarding 6PPD is its transformation into the more toxic product 6PPD-Q. The formation of 6PPD-Q via the reaction of 6PPD with ozone is well-established in the solid phase.^1,8,23,24^ There are recent reports of 6PPD-Q production in the aquatic environment from dark^8,25^ and photochemical^13,16,26^ reactivity of 6PPD. Two of these studies used quenchers to determine the relative role of reactive oxygen species, ultimately suggesting that DOM and hydroxyl radical^16^ or ^1^O_2_ ^13^ may be playing a role.

The first aim of this study was to quantify and compare the thermal and photochemical reactivities of 6PPD and 6PPD-Q in aqueous media and extend the compound scope to include IPPD (*N*-isopropyl-*N*′-phenyl-1,4-phenylenediamine). IPPD is a similar rubber antidegradant. While 6PPD is generally viewed as the superior antidegradant with a yearly production estimate from 2001 of 130,000 tonnes,^27^ IPPD is also widely used with a yearly production estimate from 2000 of 10,000–15,000 tonnes.^28^ IPPD has also been shown to react with ozone to form a quinone, with both IPPD and IPPD-quinone present in many environmental compartments, although the toxicological effects are yet to be fully studied.^10,26,29^ A recent study reported that while 6PPD-Q had toxic effects on both Coho salmon CSE-119 cell lines and juvenile rainbow trout, IPPD-quinone did not affect either.^30^ Although further studies need to be conducted on live Coho salmon and other species to rule out any toxic effects, the study posed IPPD as a potential alternative to 6PPD. The reaction rate constants of these compounds (6PPD, IPPD, and 6PPD-Q) via three routes were determined: (1) dark (thermal or ground state oxygen reactions), (2) direct photochemistry, and (3) indirect photochemistry. To our knowledge, this is the first study to separate the effects of UVA and UVB light on 6PPD photoreactivity. The second aim of this study was to determine each pathway’s 6PPD-Q molar yield. Finally, 6PPD’s reaction with ozone in the aqueous and solid-state phases was also explored. Taken together, these results present an overview of the relative importance of multiple degradation pathways for 6PPD, IPPD, and 6PPD-Q.

## 2 Methods

6PPD experiments were performed at pH 5 and 7 as 6PPD has an environmentally relevant p*K*_a_, previously calculated as 6.73 ^31^ and 6.46,^24^ but was experimentally determined in this study to be 6.35 ± 0.02 (See Section S1 in the Supporting Information (SI)). Experiments with IPPD and 6PPD-Q were performed only at pH 7. The p*K*_a_ of IPPD was also experimentally determined in this work and found to be 6.40 ± 0.03 (SI Section S1). All experimental solutions were prepared in 12.5 mM phosphate buffer unless otherwise stated. Prior to beginning each experiment, the solutions were mixed by vortex mixing and filtered through a 0.22 μm PVDF filter to remove undissolved particulates. A materials list is supplied in SI S2.

### 2.1 Dark Oxidation Reactions

Dark reactions for 6PPD, IPPD, and 6PPD-Q were performed in foil-wrapped borosilicate test tubes placed within a 19 °C Rayonet merry-go-round photoreactor (The Southern New England Ultraviolet Company, Branford, CT). For each experiment, test compounds (2 μM) were added to the buffer, and subsamples were collected periodically for analysis.

Two additional sets of thermal degradation experiments were performed with 6PPD_pH 7_, to evaluate the dependence of the reaction rate constant on both the temperature and oxygen concentration. These experiments were conducted in a quartz cuvette held inside the temperature-controlled sample holder of a UV–vis spectrophotometer (Varian Cary 100 Bio). The growth in absorbance of the degradation product, *N*-phenyl-*p*-benzoquinone monoamine (BQMI), was monitored as a proxy for the decay of 6PPD. The first set of these spectrophotometer experiments was performed at different temperatures (19.5–60.3 °C) under an atmosphere of 98% O_2_. The second series of experiments was performed at 40.5 °C under an atmosphere of varying O_2_:N_2_ content (0–98% O_2_). The gas ratios were achieved by using a dual-channel mixed flow rotameter. The gas was initially bubbled through the liquid to achieve equilibrium, and then once the experiment began, the headspace was continually purged to maintain the desired gas ratio. The absorbance values were fit to an exponential growth function to determine the rate constants. To isolate the temperature dependence of the bimolecular rate constant, the observed pseudo-first-order rate constants were divided by the oxygen concentration at each temperature. The resulting bimolecular rate constants were analyzed according to Eyring transition state theory,^32^ providing the reaction’s enthalpy of activation, Δ*H* ^‡^, and entropy of activation, Δ*S* ^‡^. Further details and equations are in SI S3.

### 2.2 Direct Photochemical Reactions

The direct photoreactions for 6PPD, IPPD, and 6PPD-Q were performed in borosilicate test tubes within a Rayonet photoreactor at 19 °C. Direct photodegradation rates were determined under both UVA (six 365 nm-centered bulbs) and UVB (two 315 nm-centered bulbs) radiation (spectra in SI S4). The direct photochemical quantum yields were determined through comparison to the photochemical reaction of the chemical actinometer, *p*-nitroanisole/pyridine (10 μM/0.5 mM), in MQ water.^33^

### 2.3 Indirect Photochemical Reactions

The reactivity of 6PPD, IPPD, and 6PPD-Q with two photochemically produced intermediates, ^3^CDOM* and ^1^O_2_, was explored by using both steady-state and time-resolved techniques. Indirect photochemistry was explored first in solutions of DOM, which produces many reactive species, including ^3^CDOM* and ^1^O_2_. To further study the contributions of ^1^O_2_, experiments were performed with well-defined ^1^O_2_ sensitizers.

#### 2.3.1 DOM

DOM solutions (5 mg_C_/L) were prepared from two DOM isolates (IHSS), Mississippi River natural organic matter (MRNOM) and Suwannee River natural organic matter (SRNOM). MRNOM is isolated from an urban watershed (Twin Cities, Minnesota) that is derived from both microbial and terrestrial precursors.^34^ SRNOM is terrestrially derived from the more pristine Okefenokee National Wildlife Refuge.^34^ SRNOM is a widely used isolate and was included for better comparison to other studies, and MRNOM was included to broaden the application of the results to other water sources. Experiments were performed under two conditions, buffer solution under ambient air atmosphere (21% O_2_) and 5% O_2_-purged buffer solution, to manipulate the ratio of the photochemically produced ^3^CDOM* present in solution. The lifetime of triplet excited states is longer at lower oxygen concentrations.^20^ These experiments were performed in a Rayonet photoreactor at 19 °C.

#### 2.3.2 Laser Experiments

A time-resolved laser system was utilized for two experiment types: ^1^O_2_ phosphorescence and transient absorption (TA) spectroscopy. Briefly, samples were irradiated with pulsed laser light (∼100 fs fwhm pulses) with variable excitation wavelengths based on the peak absorbance of the chemical of interest.

Two sets of ^1^O_2_ phosphorescence experiments were performed, following the phosphorescence signal produced from photochemically generated ^1^O_2_. First, Zn­(II)­meso-tetra­(*N*-methyl-4-pyridyl)­porphine tetrachloride (ZnP) was used as the sensitizer for the production of ^1^O_2_. Quenching of the ^1^O_2_ phosphorescence signal by 6PPD and IPPD was performed, and the pseudo-first-order rate constants obtained from these experiments were used to determine the ^1^O_2_ bimolecular quenching rate constants for each compound. Second, MRNOM or SRNOM was used as the sensitizer to produce ^1^O_2_. Quenching of the ^1^O_2_ phosphorescence signal by 6PPD or IPPD was used to obtain their respective ^3^CDOM* bimolecular quenching rate constants. The total ^1^O_2_ quenching rate constant, *k*_tot_^Δ^, is the sum of physical quenching (*k*_phys_^Δ^) and chemical reaction (*k*_chem_^Δ^). We assessed *k*_chem_^Δ^ using steady-state experiments (SI S16) and found it to be insignificant compared to that of *k*_tot_^Δ^. Accordingly, *k*_tot_^Δ^ ≈ *k*_phys_^Δ^.

To determine the nature of the reaction between ^3^CDOM* and PPD, TA experiments were performed to identify the transient species formed during the reaction. DOM was excited at 365 nm in the presence of either 6PPD or IPPD. The growth and decay of a signal at 550 nm was observed. This signal is attributed to the PPD^•+^ species, based on its similarity to the radical cation observed in spectroelectrochemical studies in acetonitrile.^35^ This suggests that the single electron transfer is the first step in the reaction.

Further explanations of the analysis are in SI S5. Due to low PPD water solubility, all experiments to determine the ^1^O_2_ and ^3^CDOM* bimolecular quenching rate constants were performed in 50/50 ACN/phosphate buffer to obtain high enough PPD concentrations to be observed in the laser system. Although the water solubilities of 6PPD and 6PPD-Q are low, estimated to be 563 ± 204 and 67 ± 5 μg/L at 23 °C,^25^ the aquatic degradation pathways are still significant as their LC_50_ for Coho Salmon toxicity are much lower, estimated at 250 μg/L^1^ and 95 ng/L,^36^ respectively.

#### 2.3.3 ^1^O_2_: Thermal Production and Self-Sensitization

Steady-state photochemically based methods for studying the reaction of 6PPD with ^1^O_2_ proved difficult due to both the competitive dark (thermal) and direct photodegradation pathways. Instead, a nonphotochemical approach was used based on the thermal decomposition of the endoperoxide, 3-(1,4-epidoxy-4-methyl-1,4-dihydro-1-naphthyl)­propanoate (MNPO_2_), which releases ^1^O_2_.^37^ To achieve different ^1^O_2_ concentrations, multiple experiments were performed with varied ratios of D_2_O and H_2_O as the solvent. The decay rate constant of ^1^O_2_ is 17.9 times slower in pure D_2_O than in H_2_O and varies linearly with the mole fractions of each.^38^ Accordingly, higher steady-state ^1^O_2_ concentrations were achieved when the reaction was performed with higher D_2_O levels. Experiments were performed in amber glass vials and purged with N_2_ to minimize the presence of light and oxygen and effectively diminish the direct photochemical and dark degradation pathways. Further details are in SI S6.

A recent study posited that 6PPD could react with ^1^O_2_ via self-sensitization.^13^ To investigate this, we monitored for the formation of ^1^O_2_ by time-resolved phosphorescence measurements. 6PPD (85 μM or diluted) was directly excited at 310 nm, and the ^1^O_2_ phosphorescence signal was collected. Perinaphthenone was employed as a reference sensitizer with a known quantum yield so that the ^1^O_2_ quantum yield of 6PPD could be determined, as detailed in SI S7. These experiments were performed in 100% buffer as well as 50/50 ACN/pH 7 buffer to facilitate better signal-to-noise ratios.

### 2.4 6PPD-Q Production

All 6PPD degradation experiments (dark, direct, and indirect) were tested for 6PPD-Q formation via HPLC-QQQ. The maximum molar yield observed for each experiment is reported (calculated as moles of 6PPD-Q produced over moles of 6PPD lost). To test the reaction of 6PPD with ozone, ozone was generated and bubbled through MQ water. A 0–10 μM dose of this ozone was then added to the reaction vessel containing 6PPD (2 μM) in buffer with stirring. Subsamples of the solutions were then frozen until analysis via HPLC-QQQ. To obtain a positive control and compare our aqueous yields to solid-state 6PPD-Q molar yields, 6PPD-coated glass slides were exposed to an ozone chamber and analyzed for 6PPD-Q production. Details are given in SI S8.

### 2.5 Sample Analysis

All steady-state photochemistry experiment samples were analyzed by HPLC-UV. About 5–10 subsamples (∼150 μL) were analyzed for each experiment. The 6PPD experiments showed similar degradation product peaks for all conditions tested. HPLC-Orbitrap MS analysis of the reaction mixtures was performed to identify the products and to better understand the reaction mechanism. HPLC and MS details are provided in SI S9.

## 3 Results and Discussion

### 3.1 Dark Oxidation Reactions

Both 6PPD and IPPD are susceptible to dark oxidation reactions. 6PPD oxidation kinetics were studied at pH values of 5 and 7, and IPPD oxidation kinetics were determined at pH 7. Comparing the kinetic data collected at 19 °C (Table 1), the dark oxidation reactivity increases in the following order: 6PPD_pH 5_ (0.42 × 10^–5^ s^–1^) ≪ IPPD_pH 7_ (2.9 × 10^–5^ s^–1^) < 6PPD_pH 7_ (4.5 × 10^–5^ s^–1^). The large difference between pH values of 5 and 7 for 6PPD is explained by the chemical structures of the neutral and anionic forms. Above its p*K*_a_ value of 6.35, 6PPD is deprotonated, more electron-rich, and therefore more easily oxidized. This is directly reflected in the order of magnitude difference between the rate constants of 6PPD_pH 5_ and 6PPD_pH 7._

**Table 1:** Temperature-Dependent Reaction Rate Constants of 6PPD_pH 7_ Dark Degradation and Dark Reaction Rate Constants for 6PPD_pH 5_, IPPD_pH 7_, and 6PPD-Q at 19 °C^a^

	pH	*T* (°C)	*k* (10^–5^ s^–1^)	*t*1/2 (h)
6PPD	5	19	0.42 ± (0.01)	46
	7	19	4.5 (±0.1)	4.3
		19.5	8.0 (±0.9)	2.4
		32.1	15 (±1)	1.3
		40.5	25 (±1)	0.76
		50.7	38 (±2)	0.50
		60.3	57 (±4)	0.34
IPPD	7	19	2.9 ± (0.1)	6.6
6PPD-Q	7	19	<0.07	>270

a Temperatures were held within approximately ±0.5 °C of the desired value.

The 1.6-fold difference between 6PPD and IPPD is less readily explained, given how structurally similar the two molecules are. The p*K*_a_ values of 6PPD and IPPD were measured to be 6.35 ± 0.02 and 6.40 ± 0.03 (SI S1), respectively. The nearly identical p*K*_a_ values result in nearly identical speciation, with 6PPD having 82% anionic form present, only 2 percentage points higher than IPPD (80% anionic) at pH 7, ruling out p*K*_a_ differences being the origin of the reactivity difference.

We hypothesize two potential causes for the reactivity difference between 6PPD and IPPD, dependent on whether the reaction rate is determined by an initial electron transfer reaction or a subsequent hydrogen atom abstraction reaction. If we assume the rate-determining reaction step is an outer-sphere electron transfer, according to Marcus theory,^39^ the two parameters determining reactivity are the one-electron oxidation potential and the reorganization energy of the reaction. The one-electron oxidation potentials of 6PPD and IPPD are both reported as −0.12 V vs Fc/Fc^+^ (0.33 vs SHE) in acetonitrile,^35^ and in this work, we also measured extremely similar oxidation potentials for the two compounds, 0.26 V vs Ag/AgCl in pH 7 water (0.47 vs SHE) (SI S10). This leaves the reorganization energy as a possible cause of reactivity differences. Outer-sphere reorganization energy is inversely dependent on the radius of the molecule.^40^ Since IPPD is smaller than 6PPD, the reorganization energy would be greater, and thus, the reaction rate would be slower.

However, if one-electron oxidation occurs with the same rate constant for both 6PPD and IPPD, then a downstream reaction could still explain the difference in the observed reactivity. For example, following one-electron oxidation, the rate of abstraction of α-hydrogen atom from the alkyl group could determine the overall rate by controlling the efficiency of the forward reaction toward products or backward repair. The order of antioxidant effectiveness for several PPDs was shown to be inversely proportional to the dissociation energy of the α-C–H bond of the alkylamine moiety.^41^ The antioxidant effectiveness of 6PPD was found to be greater than that of IPPD (277 vs 177 kg/mol at 180 °C).^41^ At the present time, we do not have enough information to judge which of these two explanations is more likely to be the cause of the rate differences.

The facile nature of the dark oxidation reaction is in agreement with many 6PPD studies, which reported half-lives ranging from 4.8 to <24 h in pH 7 buffered, tap, or DI water,^17,25,27,42^ but in contrast to two recent studies, which reported negligible dark degradation at pH 7 over 3 and 6 h.^13,16^ Although our testing conditions had significant ionic strength (12.5 mM phosphate buffer), control experiments in MQ showed extremely similar reactivity (SI S11), suggesting that ionic strength is not the cause of disparity.

In contrast to 6PPD, 6PPD-Q was stable during a 30 h observation period, as shown in Table 1. The stability of 6PPD-Q has previously been reported for even longer, as one study observed no depletion over 3 days and only 26% depletion after 47 days.^43^

The dark oxidation reactivity of 6PPD at pH 7 was further investigated. Both the oxygen concentration and temperature were found to play a significant role in the dark reactions for 6PPD_pH 7_. For this portion of the study, we determined the kinetics of 6PPD oxidation by following the formation of its oxidation product, *N*-phenyl-*p*-benzoquinone monoamine (BQMI), via its characteristic visible absorption band (eq 1).
1

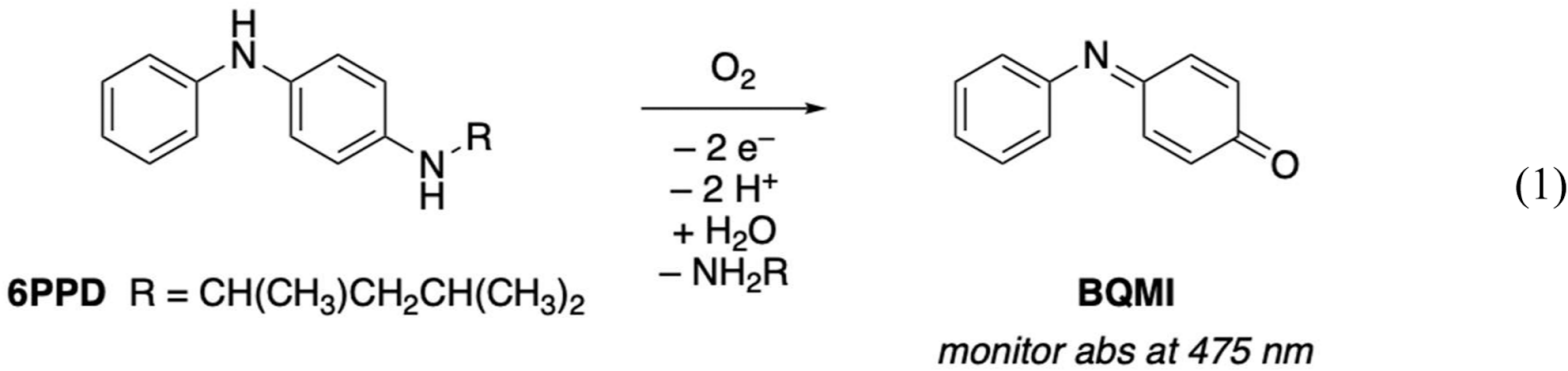

By tracking of the growth of the absorbance at 475 nm, which corresponds to the production of BQMI (Figure 1A), the decay rate constant of 6PPD could be elucidated. The observed pseudo-first-order rate constants for the formation of BQMI showed a linear relationship with oxygen concentration (Figure 1B,C), suggesting that 6PPD undergoes a bimolecular reaction with molecular oxygen.

**Figure 1:**
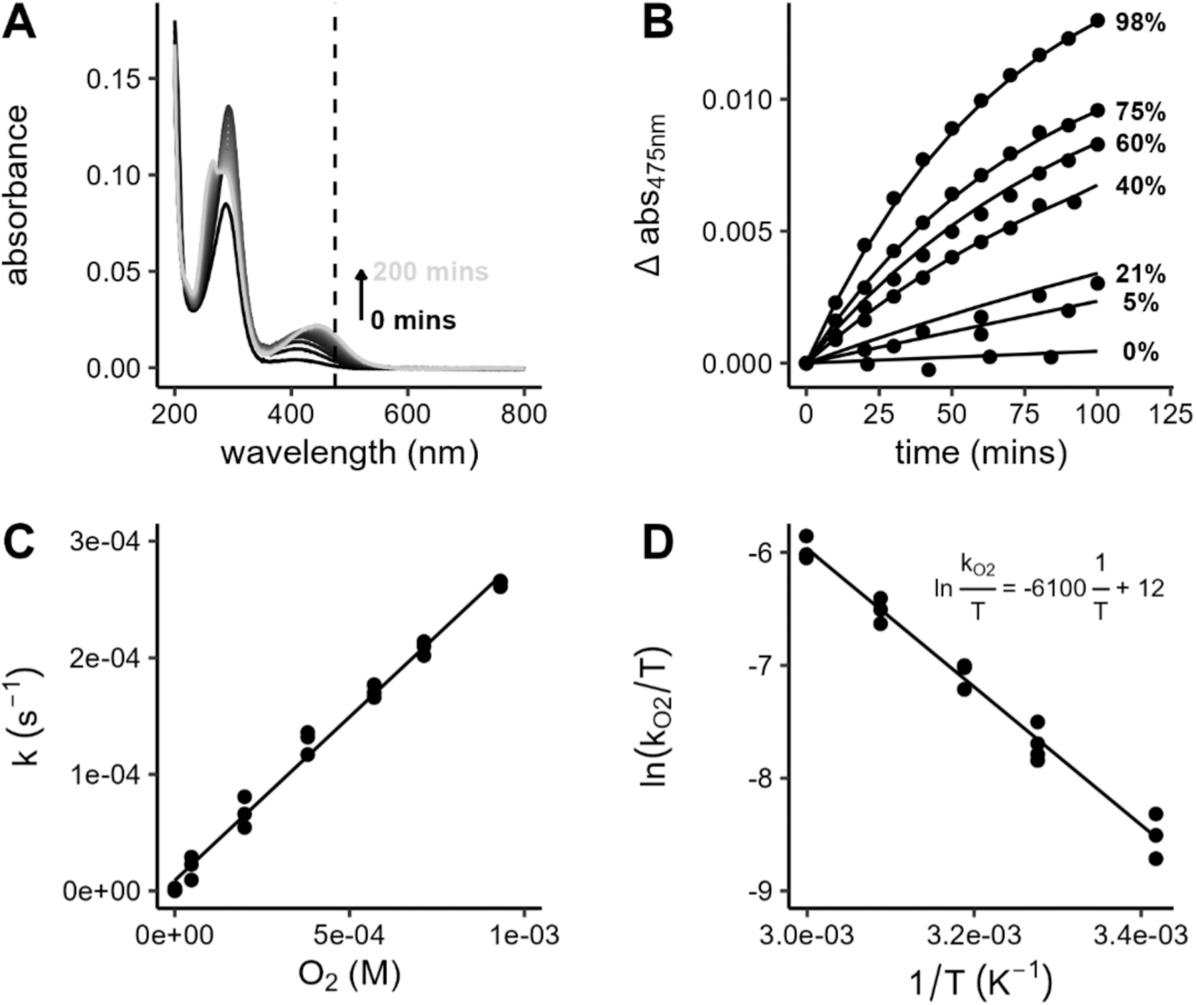
Oxygen dependence of 6PPD_pH 7_ dark degradation (performed at 40.5 °C). (A) Sample set of UV–vis spectra showing the growth of the 6PPD degradation product at 475 nm. (B) Change in 475 nm absorbance over time for oxygen levels of 0–98% O_2_. (C) Linear relationship between the 6PPD_pH 7_ dark degradation rate constant and the O_2_ concentration. (D) Eyring plot for 6PPD_pH 7_ dark degradation (performed at 98% O_2_). The linear equation is used to determine the activation parameters, enthalpy, and entropy of activation.

The Eyring plot for the temperature dependence of 6PPD_pH 7_ dark degradation, corrected for the temperature-dependent change in oxygen concentration, provides the reaction’s activation parameters (Figure 1D). The enthalpy of activation, Δ*H* ^‡^, was found to be 50.9 (±1.9) kJ mol^–1^, and the entropy of activation, Δ*S* ^‡^, was −94.4 (±6.0) J K^–1^ mol^–1^. This large negative entropy of activation is consistent with a bimolecular reaction between 6PPD and O_2_. These results suggest that the temperature and oxygen saturation of the aquatic environment will greatly affect the longevity of PPDs in the environment, with faster degradation in warm, oxygen-rich environments.

### 3.2 Photochemical Reactions

#### 3.2.1 Direct Photochemistry

First, considering the reactivity of 6PPD_pH 7_, Table 2 and Figure 2 show that the direct UVA-induced photochemical reaction is a minor contributor (20% of the total) compared to the dark reaction (the remaining 80%) at ambient 21% O_2_ conditions. However, under suboxic (5% O_2_) conditions, the dark pathway is slowed sufficiently to allow the direct UVA pathway to play a more prominent role at 56% of the total degradation. In addition to the thermal degradation being slowed under 5% O_2_ conditions, the direct degradation rate also increases in comparison to 21% O_2_ conditions, suggesting that the excited-state 6PPD reactivity also has a dependency on oxygen. It has been observed with transient absorption studies that the excited triplet state of 6PPD is quenched by O_2_.^16^

**Figure 2:**
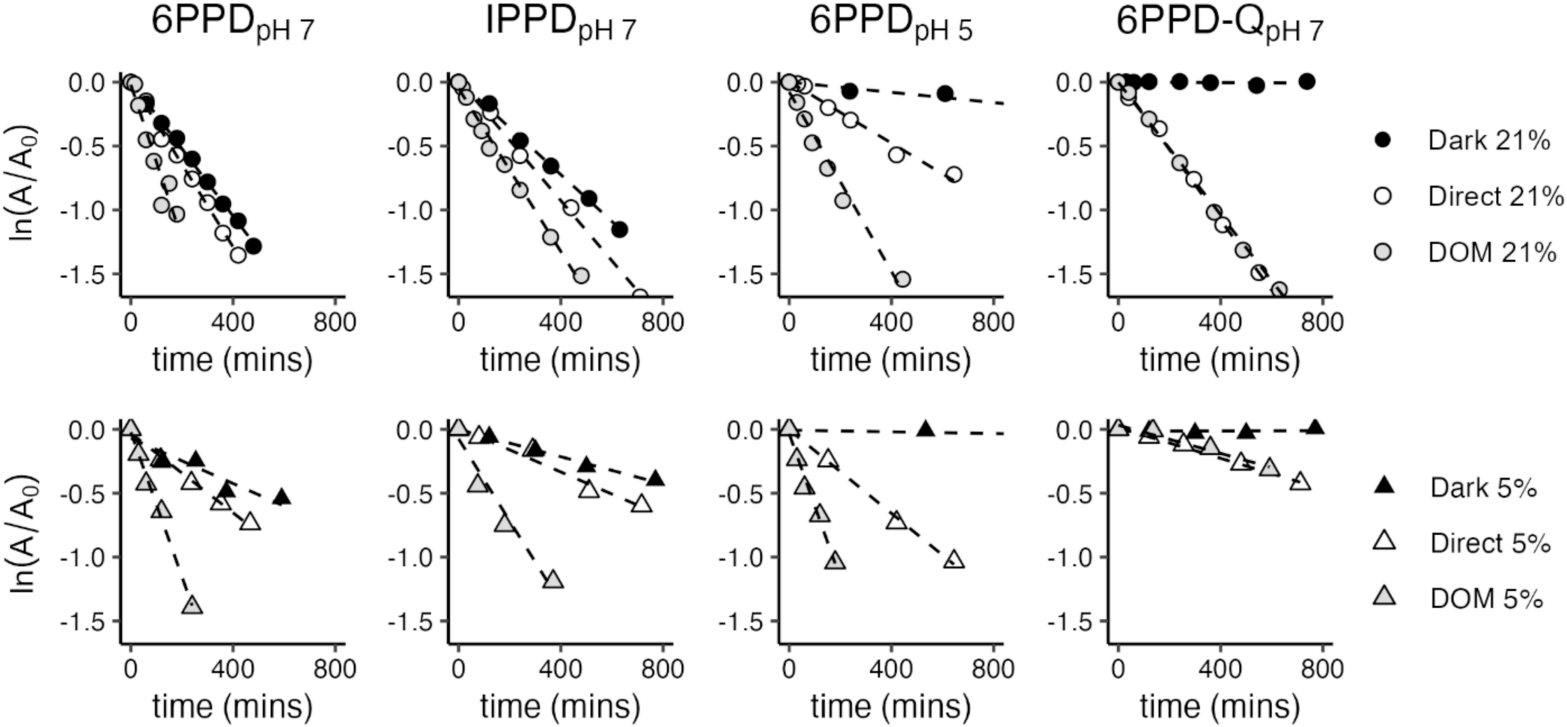
Reaction of 6PPD_pH 7_, IPPD_pH 7_, 6PPD_pH 5_, and 6PPD-Q_pH 7_ in the dark, under direct UVA irradiation, and with the addition of 5 mg_C_/L MRNOM (DOM). These experiments are shown for both 21% and 5% O_2_ conditions.

**Table 2:** Reaction Rate Constants for Dark, UVA, and UVB Reactions of 6PPD_pH 5_, 6PPD_pH 7_, IPPD_pH 7_, and 6PPD-Q_pH 7_ ^a^

	solvent	dark *k* (10^–5^ s^–1^)	UVA *k* (10^–5^ s^–1^)	UVB *k* (10^–5^ s^–1^)	UVA Φ (%)	UVB Φ (%)
6PPD_pH 5_	21% O_2_	0.42 (±0.01)	1.8 (±0.0)	85 (±5)	0.28 (±0.01)	3.3 (±0.2)
	5% O_2_	0.13 (±0.02)	2.5 (±0.1)			
6PPD_pH 7_	21% O_2_	4.5 (±0.1)	1.1 (±0.3)	23 (±1)	0.06 (±0.02)	0.92 (±0.04)
	5% O_2_	1.5 (±0.1)	1.9 (±0.6)			
IPPD_pH 7_	21% O_2_	2.9 (±0.1)	1.0 (±0.1)	12 (±0.2)	0.16 (±0.02)	0.46 (±0.01)
	5% O_2_	0.92 (±0.04)	0.58 (±0.05)			
6PPD-Q_pH 7_	21% O_2_	<0.07	4.9 (±0.4)	0.44 (±0.03)	0.004 (±0.000)	0.01 (±0.00)
	5% O_2_	<0.3	1.0			
6PPD (neutral)	21% O_2_	0.19	1.8	88	0.33	3.2
	5% O_2_	0.05	2.5			
6PPD^–^ (anion)	21% O_2_	5.5	0.9	8.4	0.05	0.34
	5% O_2_	1.8	1.8			

a UVA and UVB rate constants have the dark component subtracted. UVA and UVB quantum yields are also reported. The speciated reaction rate constants for the protonated and deprotonated forms of 6PPD were calculated by using the pH-dependent reaction rates and the 6PPD p*K*_a_ value of 6.35.

The degradation of 6PPD_pH 7_ is much faster in UVB light compared to UVA light. The quantum yields were determined to be 0.92% (UVB) and 0.06% (UVA). These values agree reasonably well with a study that reported a quantum yield of 1.14% using a xenon solar simulator lamp as the light source.^16^ Faster degradation under UVB irradiation occurs both because of the higher quantum yield and because 6PPD, with a peak absorbance around 290 nm, absorbs light more efficiently in the UVB region (molar absorbance spectra for all chemicals in SI S12). Photochemical rate constants are presented in Table 2; the corresponding kinetic traces for 6PPD as well as the other investigated phenylenediamines can be found in SI S13.

The direct photodegradation trends were similar for the IPPD_pH 7_ and 6PPD_pH 5_. IPPD reacted slightly slower than 6PPD. This is likely due to the same factors controlling the dark reactivity, as discussed above. Under our photochemical conditions, direct photodegradation of IPPD accounts for 26% of the degradation under 21% O_2_ (ambient) conditions, and this increases to 39% of the total degradation rate under 5% O_2_ conditions. Again, UVB irradiation results in more efficient photodegradation than UVA, with the UVA quantum yield determined to be 0.16% and UVB determined to be 0.46%. One difference between 6PPD and IPPD is that the IPPD direct degradation rate constant decreased with decreasing oxygen concentration, while 6PPD increased. This suggests that triplet excited IPPD may not be as reactive with oxygen as 6PPD.

The reactivity of 6PPD was examined at pH values of 5 and 7. 6PPD_pH 5_ showed similar behavior to 6PPD_pH 7_; however, direct photochemistry plays a more important role because the dark reactions are much slower in comparison to 6PPD_pH 7_. Both the UVA and UVB reaction rates for 6PPD are faster at pH 5, and this is likely due to the higher quantum yields of 6PPD in its protonated state. The quantum yields for 6PPD_pH 5_ are 0.28% in UVA and 3.3% in UVB, both higher than that of 6PPD_pH 7_. Regarding the role of oxygen in direct photochemistry at pH 5, we again observed an increase in the rate of direct photoreaction under 5% O_2_ conditions. The direct photochemistry contribution increases from 81% under ambient oxygen conditions to 95% under suboxic (5% O_2_) conditions. Figures comparing 21% O_2_ and 5% O_2_ conditions on the same plot for each chemical are shown in SI S14.

Using the 6PPD p*K*_a_ value of 6.35 and the pH-dependent kinetic data, we were able to calculate the speciated reaction rate constants for the neutral and anionic forms of 6PPD. These values are reported in Table 2 for 6PPD reactivity in the dark (5% O_2_ and 21% O_2_), direct UVA light (5% O_2_ and 21% O_2_), and direct UVB light (21% O_2_). These values allow for the estimation of the reaction rate at any pH value. The quantum yield values for the pure 6PPD (neutral) and 6PPD^–^ (anion) components were also calculated (Table 2). While similar to each other, the neutral species has a higher quantum yield compared with the anionic species for both UVA and UVB wavelengths. This could be due to either the excited-state anion having more efficient deactivation than the excited-state neutral species or the excited-state anion being less reactive than the excited-state neutral. We tentatively favor the former explanation, as it seems less likely that the excited-state anion would be less reactive toward follow-up oxidation processes than the excited-state neutral species.

As mentioned previously, 6PPD-Q is stable under dark conditions but undergoes direct photochemical degradation, as shown in Figure 2. Under UVA light, a degradation rate constant of 4.9 ± 0.4 × 10^–5^ s^–1^ was observed, faster than those of both 6PPD and IPPD. 6PPD-Q has an absorption peak around 366 nm, aligning well with the 365 nm-centered UVA bulbs. One observation of note is that, opposite to 6PPD, decreasing the oxygen content decreases the direct photochemical reaction rate, suggesting that the excited state of 6PPD-Q may react with oxygen. Under UVB light (centered closer to the 6PPD and IPPD max absorption), 6PPD-Q degrades more slowly than 6PPD and IPPD, with a rate constant of only 0.44 ± 0.03 × 10^–5^ s^–1^. The only other study reporting a 6PPD-Q direct photochemical rate constant found an average degradation rate constant of 1.1 × 10^–5^ s^–1^ using a 20 °C high-latitude solar simulator, comparable to the values measured in the present study.^21^ The prior study also reports that the direct photochemical reactivity of 6PPD-Q is temperature-dependent, something that was not further investigated here. We again found the quantum yield to be wavelength-dependent, with a UVA quantum yield of 0.004% and UVB of 0.010%, more than an order of magnitude lower than 6PPD or IPPD. These values agree with the previously reported quantum yield for 6PPD-Q of 0.0015% at 20 °C under simulated high-latitude sun.^21^

#### 3.2.2 Indirect Steady-State Photochemistry: DOM

The presence of 5 mg_C_/L Mississippi River Natural Organic Matter (MRNOM) increases the photodegradation rate compared to direct photolysis for 6PPD_pH 7_, IPPD_pH 7_, and 6PPD_pH 5_ under ambient (21% O_2_) and suboxic (5% O_2_) conditions (Figure 2). Under 5% O_2_ conditions, based on previously measured values for the oxygen-dependent and -independent rate constants for MRNOM triplet states,^44^ the triplet lifetime will be approximately double compared to ambient O_2_ concentrations. The degradation rate constant due to reactions with ^3^CDOM* will concomitantly double. For 6PPD_pH 7_ and IPPD_pH 7_, a distinct increase in the indirect photochemical portion of the degradation was observed, as shown by the increased gap between the direct and the DOM data in Figure 2. The reactivity does not double, meaning that there are still other species in addition to ^3^CDOM* degrading 6PPD and IPPD, but the increase is consistent with ^3^CDOM* playing an important role. The additional increased reactivity could be due to ^1^O_2_ (discussed below), or other reactive species. These non-^3^CDOM* reactive species are responsible for the indirect photochemical reactivity found for 6PPD_pH 5_, as the DOM-associated enhancement to the photochemical reaction rate constant did not increase under 5% O_2_ conditions.

6PPD-Q showed no enhancement of the photochemical rate with DOM added to the solution, indicating that indirect photochemistry plays a negligible role. In fact, the reaction rate decreased slightly with the addition of DOM due to light screening. The same is true for 5% O_2_ conditions, suggesting that the triplet pathway is likely not important. The study by Redman et al. showed that adding 10 mg/L SRFA to the solution, equivalent to the NOM concentration in this study (5 mg_c_/L = 9.9 mg/L SRNOM), did increase the degradation rate slightly, changing the half-life from 17.4 ± 4.5 h under direct conditions to 11.2 h with SRFA present. One potential explanation is that SRFA is a better sensitizer than MRNOM and SRNOM, the DOM sources used in this study. They also noticed that the degradation was greater at lower temperatures with DOM present, ultimately suggesting phenoxy or hydroxyl radicals cause the change.^21^ However, in lake water, with only 4.4 mg/L DOM, they did not see any change in degradation rate, aside from some light screening, indicating that degradation depends on the concentration and type of DOM in the waterbody.

For each of the four compounds, the same experiments were performed with SRNOM rather than MRNOM and showed very similar trends, essentially acting as a duplicate (SI S15). The variance in the DOM’s source and anthropogenic influence did not have a large impact, which expands the applicability of the results to a wider range of water sources.

#### 3.2.3 Quenching of ^3^CDOM* and ^1^O_2_ by 6PPD and IPPD

The bimolecular rate constants with ^3^CDOM* (*k*_tot_^T^) and ^1^O_2_ for both MRNOM and SRNOM with 6PPD and IPPD were determined through ^1^O_2_ phosphorescence. A transient absorption system was used to monitor the formation of transient species to help determine the reaction mechanism.

An example set of data from each time-resolved system is shown in Figure 3. The plots from other time-resolved experiments can be found in SI S17 and S18. On the left side of Figure 3, the ^1^O_2_ phosphorescence growth and decay curves for 6PPD and MRNOM are shown. The most intense curve is the ^1^O_2_ signal from the MRNOM alone. As the 6PPD concentration increases, the ^3^CDOM* is quenched and the ^1^O_2_ signal decreases. The growth rates of these curves hold key information about *k*_tot_^T^. On the right side of Figure 3, the 3D transient absorption spectra of 6PPD (1.22 mM) and MRNOM are shown. The signal at 550 nm is attributed to the formation of the 6PPD radical cation, similar to the spectrum observed for this species in CH_3_CN by spectroelectrochemistry.^35^ The observation of this signal for both 6PPD and IPPD in the presence of DOM suggests that an electron transfer from PPD to ^3^CDOM* is the first reaction step.

**Figure 3:**
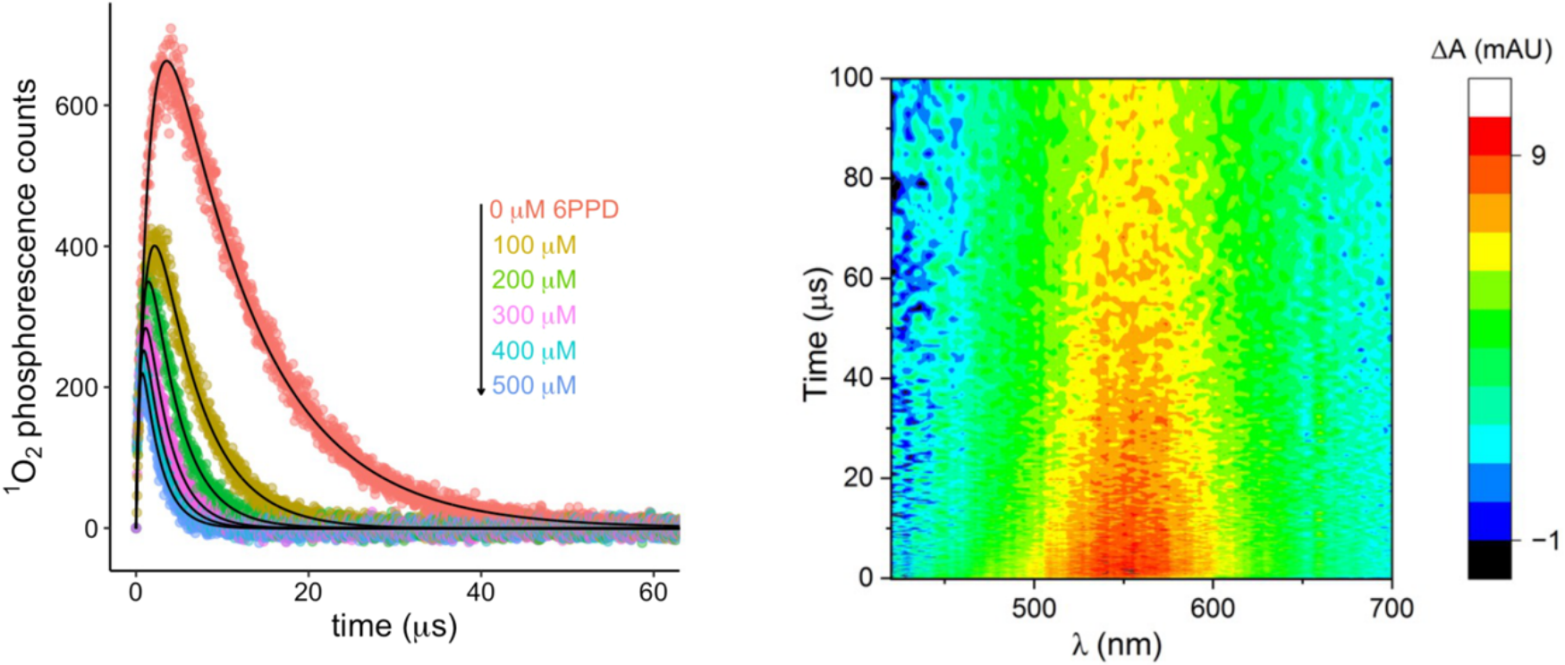
Left: Time-resolved singlet oxygen (^1^O_2_) phosphorescence traces for MRNOM upon 365 nm excitation as a function of added 6PPD. Solid lines are biexponential growth and decay fits. Right: 3D transient absorption spectra of MRNOM with the addition of 1.22 mM 6PPD upon 365 nm laser excitation, measured up to 100 μs.

6PPD_pH 7_ shows greater reactivity with ^3^CDOM* than 6PPD_pH 5_ and IPPD_pH 7_ (Table 3). The data for MRNOM and SRNOM are comparable to each other, with the two DOM sources again showing no significant differences.

**Table 3:** Bimolecular Reaction Rates of ^1^O_2_ Quenching (*k*_tot_^Δ^) and ^3^CDOM* Quenching (*k*_tot_^T^) via ^1^O_2_ Phosphorescence (^1^O_2_ Phosph)

	*k*_tot_^Δ^ (10^8^ M^–1^ s^–1^)	DOM	*k*_tot_^T^ (10^8^ M^–1^ s^–1^) ^1^O_2_ phosph
6PPD_pH 5_	12.0 (±0.4)	MRNOM	8.7 (±1)
		SRNOM	8.7 (±1)
6PPD_pH 7_	14.2 (±0.6)	MRNOM	16 (±1)
		SRNOM	14 (±3)
IPPD_pH 7_	12.1 (±0.5)	MRNOM	9.7 (±1)
		SRNOM	11 (±2)

### 3.3 6PPD-Q Formation Pathways

In our study, minimal 6PPD-Q was produced from 6PPD in aqueous solution via all explored pathways (dark, direct UVA, direct UVB, and ^3^CDOM*). All molar yields of the production of 6PPD-Q from 6PPD were less than 1%, with one exception, which was dark degradation in the presence of MRNOM in pH 5 conditions. Here, the maximum yield of 6PPD-Q was 2.5%. Another study had similar findings that the addition of DOM during light exposure caused increased transformation.^16^ Molar yields of 6PPD-Q for direct degradation have been reported as less than 1 ^16^ and 3%^13^ in aquatic environments using solar simulators. Our results for the direct molar yield are lower (0.17% maximum), but differences in the irradiation sources make direct comparisons difficult. The lamps used here are polychromatic but have a wavelength range smaller than that of solar simulators. Ultimately, molar yields may change based on the spectral range explored. As 6PPD-Q is being produced in solution, it is also simultaneously being degraded via direct photochemistry.

The reaction of 6PPD with ozone, both in solution and in the solid state, was explored. Upon addition of 2–10 μM ozone to an aqueous solution of 2 μM 6PPD, the maximum 6PPD-Q molar yield observed was 0.12% (SI S19). In comparison, the ozonation of solid-state 6PPD-coated slides resulted in a molar yield of 11.3%. This agrees with Seiwert et al., who reported 6PPD-Q peak areas five times higher during ozonation of 6PPD in the solid state versus in an aqueous solution.^8^ Recent studies reported average molar yields of 6PPD-Q from the reaction of ozone with 6PPD in the solid state of 10 ± 5 ^23^ and ∼9.7%.^24^ Our results are well-aligned with prior studies and further confirm that the production of 6PPD-Q in aquatic environments through reaction with ozone is much lower compared to the solid state.

### 3.4 6PPD-Q Formation Pathways

Based on testing across dark, direct, and indirect pathways, three main aquatic degradation products were identified via HPLC-Orbitrap MS. These products include *N*-phenyl-*p*-benzoquinone monoimine (BQMI), 4-hydroxydiphenylamine (HDPA), and *N*-(4-(1,3-dimethylbutyl)­imino)-2,5-cyclohexadiene-1-ylidene (6PPDQI), with mass-to-charge ratios ([M + H]^+^) of 184.0761, 186.0918, and 267.1856, respectively. The photochemical and nonphotochemical (dark) pathways are most likely similar, as all products were seen in both dark and photochemical reactions. The products were confirmed only for 6PPD, but we expect similar pathways for IPPD.

The proposed mechanism (Figure 4) begins with 6PPD in equilibrium with its deprotonated anion counterpart. The deprotonated 6PPD^–^ reacts more readily due to increased electron richness. We speculate that 6PPD reacts with oxygen to form the *N*-based radical, which can then further react with oxygen to form 6PPDQI. Subsequently, 6PPDQI reacts by imine hydrolysis to form BQMI, which can be subsequently reduced to HDPA. HDPA was confirmed via an analytical standard and has been reported consistently throughout the literature.^8,13,27^ BQMI was confirmed via an in-house standard and was previously reported as a 6PPD degradation product.^27^ 6PPDQI was not confirmed with a standard but is commonly reported as a hydrolysis product.^8,13,45,46^

**Figure 4:**
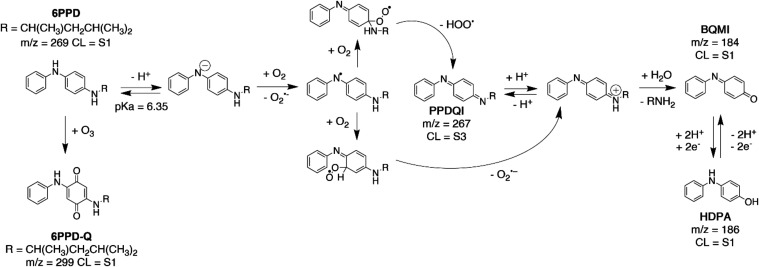
Hypothesized reaction pathway for 6PPD to products in both dark and photochemical conditions. The confidence levels (CL) of the proposed 6PPD products follow the convention proposed by Schymanski et al.^47^

## 4 Environmental Implications

This study demonstrates the relative contributions of thermal and photochemical degradation pathways for 6PPD, IPPD, and 6PPD-Q in the aquatic environment. The deprotonated forms of 6PPD and IPPD degrade facilely due to thermal reactions, with dependencies on both oxygen and temperature. Under more acidic conditions, the thermally more stable neutral 6PPD is more prevalent, and photochemical processes take on an increased importance as a degradation process. Photochemical degradation is enhanced in the presence of DOM, likely due to excited triplet-state organic matter and not due to ^1^O_2_. In general, 6PPD and IPPD readily degrade and will likely not persist in most aquatic environments. They will have greater persistence in cold, dark, and anoxic conditions. In our results, IPPD often had similar or slightly longer lifetimes than 6PPD, suggesting that it is generally less reactive. Lower reactivity is a desirable trait if IPPD is proven to be less likely to react with ozone to form its quinone (if the quinone also proves to be toxic). However, the longer lifetimes suggest that IPPD could accumulate to higher environmental concentrations, which is undesirable. Before a decision about replacing 6PPD with IPPD is made, more work is needed to test IPPD and its quinone in all environmental conditions (temperature, pH, etc.) and with exposure to a range of aquatic organisms to be sure of the chemicals’ fate and toxicity.

In comparison to 6PPD and IPPD, 6PPD-Q is expected to have a longer aquatic lifetime. 6PPD-Q showed minimal reactivity in the dark as well as in the presence of ^3^CDOM* and ^1^O_2_. The main 6PPD-Q degradation pathway is direct photochemistry, specifically under UVA light. Therefore, most degradation will occur near the water surface, and 6PPD-Q will likely be persistently lower in the water column and in sediment. However, 6PPD-Q is not formed in aqueous solution in substantial amounts through any pathway that we have examined. By contrast, ozonation of 6PPD in the solid state produces significant yields of 6PPD-Q. This suggests that the production of 6PPD-Q is likely happening within the tire rubber or on the road surface prior to transport into waterways. Therefore, 6PPD substitutions or regulations or roadside buffer remediation will likely be essential to keep 6PPD-Q out of waterways.

## Supplementary Material



## Notes

p*K*_a_ Measurements, details of materials and methods, irradiance spectra, molar absorptivity data, kinetic data, transient absorption data, and electrochemistry data (PDF)
