# Supplementary material for: Aquatic Thermal and Photochemical Reactivity of N‑(1,3-Dimethylbutyl)‑N′‑phenyl‑p‑phenylenediamine (6PPD), N‑Isopropyl‑N′‑phenyl‑p‑phenylenediamine (IPPD), and 6PPD-quinone
Source: Environ Sci Technol. 2025 Jun 16;59(25):12900–9. doi: 10.1021/acs.est.4c12896 (PMC12224295; doi:10.1021/acs.est.4c12896)
Supplement: Supplementary file 1 [file es4c12896_si_001.pdf]

Supporting Information

for

The Aquatic Thermal and Photochemical Reactivity of *N*-(1,3-dimethylbutyl)-*N'*-phenyl-*p*-phenylenediamine (6PPD), *N*-isopropyl-*N'*-phenyl-*p*-phenylenediamine (IPPD), and 6PPD-quinone

Kathryn L. Platt,[a] Oleksandr Yushchenko,[a] Juliana R. Laszakovits,[a]

Yiwen Zhang,[a] Nicholas A. Pflug,[ab] Kristopher McNeill[a*]

[a] Institute of Biogeochemistry and Pollutant Dynamics, ETH Zurich, 8092 Zurich, Switzerland

[b] Department of Chemistry, SUNY ESF, Syracuse NY, 13210 USA

*corresponding author

Pages: 32

Figures: 17

Tables: 2

Table of Contents

S1. pK_a Measurements....3
S2. Materials....3
S2a. Production of $MNPO_2$....4
S2b. Production of BQMI In-House Standard....5
S3. UV-Vis Dark Reactions Methodology....6
S4. Irradiance Spectra....7
S5. Laser Experiment Methodology....8
S6. Steady-State $^1O_2$ Methodology....9
S7. $^1O_2$ Quantum Yield Calculations....11
S8. 6PPD-Q Solid-State Ozone Methodology....11
S9. HPLC and MS Methods....12
S10. Electrochemistry of 6PPD and IPPD....14
S11. Ionic Strength Effects....15
S12. Molar Absorbance Spectra of 6PPD, IPPD, and 6-PPD-Q....16
S13. UVA vs UVB Direct Photochemistry Kinetic Data....20
S14. Dark and Direct UVA Photochemistry....21
S15. SRNOM Steady-State Kinetic Data....22
S16. Steady-State $^1O_2$....23
S17. $^1O_2$ Phosphorescence Model Fits....28
S18. Transient Absorption DOM + 6PPD....30
S19. 6PPD-Q Formation and Ozone Experiments....31
References....31

S1. pK_a Measurements

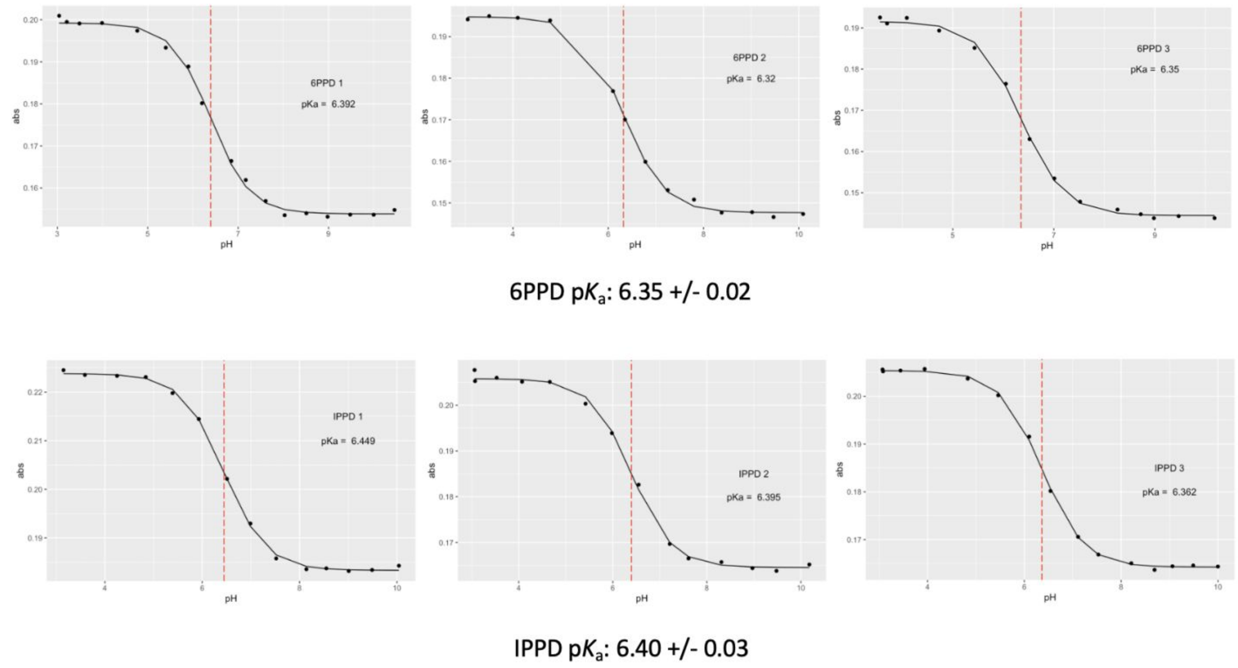

Figure S1: Triplicate pK_a measurements for 6PPD and IPPD. 6PPD or IPPD was dissolved in nitrogen-purged 0.4 mM phosphate buffer (pH 3), and NaOH was used to incrementally increase the pH. UV-vis measurements were taken at each pH increment and the 284 nm absorbance reading data was fit in R to provide the pK_a value for each. The average pK_a of triplicates was obtained for each chemical.

S2. Materials

6PPD (>98%, TCI Chemicals) and IPPD (>97%, TCI Chemicals) stock solutions were prepared in nitrogen purged ACN (≥99.9%, Sigma), stored in a nitrogen glovebox, and frequently replaced. 6PPD-Q (>97%, HPC Standards) was prepared in ACN and stored in the dark at 4°C. Either Zn(ii)meso-tetra(*N*-methyl-4-pyridyl)porphine tetrachloride (ZnP) (Sigma Aldrich) or 3-(1,4-epidoxy-4-methyl-1,4-dihydro-1-naphthyl)propanoate ($MNPO_2$) (synethesized in-house, details in S1a) was used as a well-defined source of $^1O_2$ and furfuryl alcohol (FFA) (98.0%, Thermo) as a $^1O_2$ probe. All probe and sensitizer solutions were prepared in high purity water except for ZnP which was prepared in 50/50 ACN/MQ. Two dissolved organic matter (DOM) isolates, Suwanee River natural organic matter (SRNOM) and Mississippi River natural organic matter (MRNOM), were purchased from the International Humic Substance Society, dissolved in 12.5 mM phosphate buffer (pH 5 or pH 7), and filtered with a GHP 0.2 μM filter prior to use. Actinometry experiments were performed using pyridine (99.5% Acros) and recrystallized *p*-nitroanisole (PNA) (97%, Aldrich). The identified 6PPD degradation product, 4-hydroxydiphenylamine (4-HDPA) was purchased from fluorochem, while the other degradation product, *N*-phenyl-*p*-benzoquinone monoamine (BQMI), was synthesized as described in SI S2b. All experimental solutions were prepared in 12.5 mM phosphate buffer (potassium phosphate monobasic ≥99.0%, Sigma)

S2a. Production of $MNPO_2$

The synthesis of $MNPO_2$ was performed via photochemical methods, using a hybrid of published methods.[1,2] The starting material 3-(4-methyl-1-naphthyl)propanoic acid (MNP) was

purchased from Fluorochem. MNP (38 mg) was mixed with 10 mL ACN and dissolved via sonication. Once dissolved, ZnP (10 μM) was added to the solution. The solution was placed in an ice bath and constantly stirred and purged with O_2 during light exposure from a xenon lamp (Newport, 50-500 W), with a 435 nm cutoff filter applied to prevent the direct degradation of the MNP and $MNPO_2$. The transformation of MNP to $MNPO_2$ was checked periodically via UV-vis spectroscopy and HPLC. Extra 10 μM additions of ZnP were made at 90, 155, 245, and 350 minutes to compensate for ZnP photobleaching. About 98% conversion was achieved. The solution was passed through a silica column with 30/70% ethanol/ACN which retained the positively charged ZnP on the column. The resulting solution was characterized by HPLC and UV-vis, dried via N_2 purge while cooled to 0 °C, and stored at -20 °C until use.

S2b. Production of BQMI In-House Standard

N-phenyl-*p*-benzoquinone monoamine (BQMI; m/z 184) was produced via oxidation of 4-hydroxydiphenylamine (4-HDPA).[3] 4-HDPA (20 mg) was added to ACN (3 mL) and dissolved with stirring. A 12% NaOCl solution (500 μL) was added under stirring and the reaction was allowed to proceed for 30 min at room temperature. Excess NaOCl was neutralized with sodium sulfite (ca. 150 mg) and removed by filtration. To isolate the product, water (10 mL) was added and the solution was concentrated under a N_2 stream to remove ACN. The solution was then filtered via suction filtration through an MCE filter and the collected solids were washed with water, leaving a pink-red solid.

The synthesized BQMI matched the observed 6PPD degradation product (m/z 184.0757) by mass spectrometry (m/z 184.0742), by HPLC retention time, and by its absorbance spectrum (Figure S2).

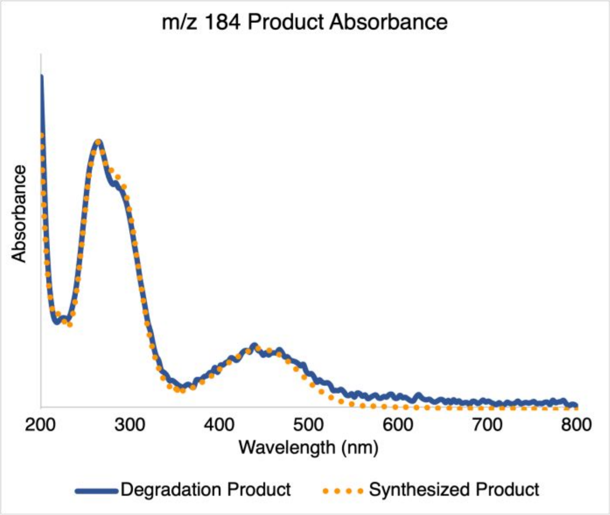

Figure S2: Comparison of the normalized absorbance spectra of the degradation product, m/z 184, observed in 6PPD degradation experiments, and synthesized BQMI.

S3. UV-Vis Dark Reactions Methodology

The changes in absorbance at 475 nm (due to the growth of BQMI from the degradation of 6PPD) over time were fit in R[4] to an exponential growth function (Equation S1).

$$Abs_t = (Abs_\infty - Abs_o) \times (1 - e^{-k \times t}) + Abs_o \quad \text{(S1)}$$

The time, t, and absorbance at time t, Abs_t, are produced from the collected data, while the absorbance at time infinity, Abs_∞, absorbance at time zero, Abs_o, and the growth rate constant, k, are determined from the model fit.

To isolate the change in rate constant due to temperature changes, the rate constant was divided by the oxygen concentration at each temperature to obtain the bimolecular reaction rate constant between 6PPD and O_2. Using Eyring transition state theory, as shown in equation S2, and the oxygen independent rate constants, $k/[O_2]$, the temperature-dependent data was used to elucidate the activation parameters, enthalpy of activation, $\Delta H^{\ddagger}$, and entropy of activation, $\Delta S^{\ddagger}$.

$$\ln\left(\frac{k/[O_2]}{T}\right) = -\frac{\Delta H^{\ddagger}}{R} \times \left(\frac{1}{T}\right) + \frac{\Delta S^{\ddagger}}{R} + \ln\left(\frac{k_b}{h}\right) \quad \text{(S2)}$$

where R is simply the universal gas constant, k_b is Boltzmann's constant, and h is Planck's constant.

For these experiments, temperature was monitored using an EL-USB thermistor probe data logger (Lascar, UK). Oxygen concentrations were monitored using a PreSens OXY-1 ST probe (Precision Sensing, Germany).

S4. Irradiance Spectra

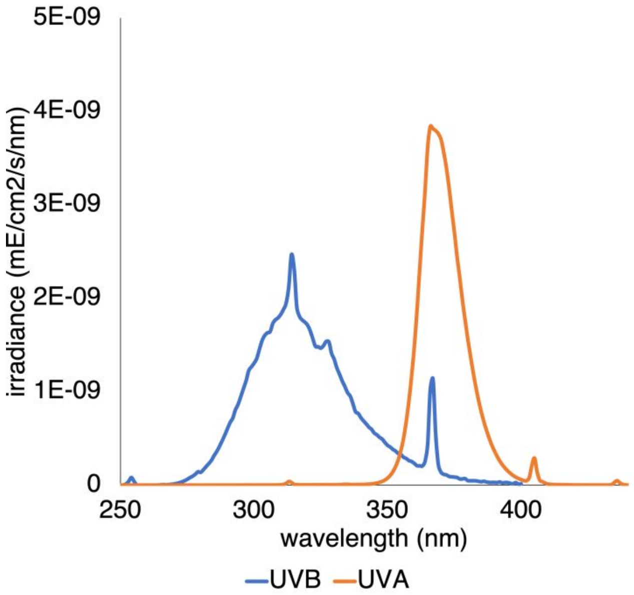

Figure S3: The relative irradiance spectra of the UVB and UVA bulbs used in the Rayonet photoreactor in steady-state photochemical experiments.

S5. Laser Experiment Methodology

The laser setups have both been previously described.[5] As previously shown, $^1O_2$ phosphorescence can be used as a proxy for ^{3}CDOM* reactivity, as $^1O_2$ is created via energy transfer from ^{3}CDOM* to O_2.[5] To more accurately elucidate the bimolecular rate constant between 6PPD or IPPD and ^{3}CDOM*, first having a good estimate of the $^1O_2$ bimolecular rate constant is desirable to limit the number of unknowns in the fit of the $^1O_2$ growth-decay curve. To obtain this, ZnP (5 μM) was excited at 440 nm and the time-resolved $^1O_2$ signal was collected. Various concentrations of either 6PPD or IPPD (0-1000 μM) were added to quench the

$^1O_2$ signal. The slope of the Stern-Volmer plots of the decay rate constant versus concentration is equal to the PPD-$^1O_2$ bimolecular quenching rate constant, k_{tot}^{Δ}.

$$k_d = k_{tot}^{\Delta} \times [PPD] + k_d^{\Delta} \tag{S3}$$

k_d is the observed $^1O_2$ decay rate and k_d^{Δ} is the pseudo-first order rate constant for $^1O_2$ nonradiative deactivation due to the solvent. The $^1O_2$ biomolecular quenching rate constant was then used in the model to fit the growth and decay of the signal formed from a DOM + PPD quenching experiment. Here, MRNOM (90 mg/L) or SRNOM (75 mg/L) was excited at 365 nm and various concentrations of 6PPD or IPPD were added to quench the $^1O_2$ phosphorescence signal formed. The slope of the Stern-Volmer plots of the *growth* rate constant versus concentration is equal to the PPD-^{3}CDOM* bimolecular quenching rate constant, k_{tot}^{T}.

$$k_f = k_{tot}^{T} \times [PPD] + k_{O_2}[O_2] + k_d^{T} \tag{S4}$$

Where k_f is the observed $^1O_2$ formation rate, k_{O2} is the ^{3}CDOM-O_2 quenching rate, and k_d^{T} is the pseudo-first-order rate constant for all ^{3}CDOM* deactivation processes other than quenching by PPD or O_2. For all $^1O_2$ experiments, kinetic traces collected from argon-purged solutions were subtracted to remove any background luminescence counts. All data was fit in R.

For transient absorption experiments, MRNOM (90 mg/L) or SRNOM (75 mg/L) was excited at 365 nm and various concentrations of 6PPD or IPPD were dosed in to quench the ^{3}CDOM*. The growth of a signal at 550 nm, assigned to the PPD radical cation, was observed.

S6. Steady-State $^1O_2$ Methodology

Initially, the known $^1O_2$ sensitizer, ZnP, was employed in Rayonet experiments at a concentration of 0.5 μM (and also 4 μM for 6PPD-Q which showed little reactivity). These experiments were performed in the Rayonet photoreactor at 19 °C, but with two test tubes side-by-side, one containing ZnP and the compound of interest (6PPD or 6PPD-Q), and the other containing ZnP and the $^1O_2$ probe (FFA). Four UVA bulbs were used to excite ZnP and produce $^1O_2$. The PPD solutions were filtered prior to adding the sensitizer. FFA was employed to measure the steady-state concentration of $^1O_2$. This experiment was performed at normal buffer conditions, as well as in 50% D_2O to manipulate the lifetime of $^1O_2$ in solution. The presence of 50% D_2O should result in approximately 50% greater steady-state $^1O_2$ concentrations due to slower relaxation times in D_2O vs H_2O. Therefore approximately 50% faster degradation of the FFA or PPD is expected with D_2O if $^1O_2$ is the prominent degradation pathway.

As the PPDs were found to undergo dark and direct degradation, a method to remove these pathways and focus solely on the $^1O_2$ pathway was desirable. In order to accomplish this, the endoperoxide, 3-(1,4-epidoxy-4-methyl-1,4-dihydro-1-naphthyl)propanoate ($MNPO_2$), was used as a well-defined source of $^1O_2$. A solution of FFA (20 μM) in D_2O was heated in an amber glass vial to 37 °C and purged with N_2 to minimize the presence of light and oxygen. When heated, the endoperoxide releases $^1O_2$ into solution where it can react with the FFA and 6PPD. Once the temperature and O_2 concentration stabilized, 6PPD (2 μM) and $MNPO_2$ (1 mM) were spiked into solution and aliquots were taken every 11 minutes for immediate HPLC analysis of FFA and 6PPD. D_2O was used to limit the concentration of $MNPO_2$ required. To manipulate the $^1O_2$ concentration, an identical experiment was performed with only 50% D_2O and 50% pH 7 phosphate buffer, where the steady-state $^1O_2$ concentration is expected to be lower and therefore would lower degradation if $^1O_2$ reactivity is a prominent pathway.

S7. $^1O_2$ Quantum Yield Calculations

The singlet oxygen quantum yields (Φ_Δ) were calculated with the following formula using the data from the sensitizer of interest (Sens) was 6PPD and the reference sensitizer (Ref) was perinaphthenone:

$$\Phi_\Delta(\text{Sens.}) = \Phi_\Delta(\text{Ref.}) \times \frac{s_\Delta(\text{Sens.})}{s_\Delta(\text{Ref.})} \times \frac{R_{\text{abs}}\ (\text{Ref.})}{R_{\text{abs}}\ (\text{Sens.})} \times \frac{\text{Coll.}\ t\ (\text{Ref.})}{\text{Coll.}\ t\ (\text{Sens.})}$$

$$R_{\text{abs}} = \frac{\sum_\lambda I_{\lambda,\text{rel}}(1 - 10^{-\alpha_\lambda l})}{l}$$

where s_Δ is the area under the $^1O_2$ growth-decay curve, R_{abs} is the rate of light absorbance (mmol photons cm^{-3} s^{-1}), and Coll. t is the amount of time the signal was collected for (s). $I_{\lambda,rel}$ is the relative emission intensity of the laser excitation measured with a Jaz radiometer (Ocean Optics) over 1 nm wavelength intervals (counts), α_λ is the decadic absorbance coefficient of the solution at wavelength λ (cm^{-1}) and l is the pathlength of the cuvette (1 cm).

S8. 6PPD-Q Solid-State Ozone Methodology

To obtain a positive control and compare our aqueous yields to solid-state 6PPD-Q molar yields, 6PPD-coated glass slides were placed in a Bioforce Nanosciences UV-ozone chamber Glass slides were coated with a 0.4 mM 6PPD stock (applied in ACN and then left to dry for 30 min). These slides were then placed inside the ozone-filled chamber, shielded from the light.

Two controls were used, one covered in a second glass slide to prevent ozone flow and one covered with a second covered glass slide to prevent ozone and light penetration. The slides were extracted with ACN via sonication and then analyzed via HPLC-MS for 6PPD-Q.

S9. HPLC and MS Methods

The following abbreviations are used in this section (MQ = MilliQ Water, ACN = Acetonitrile, MeOH = Methanol, FA = Formic Acid). HPLC and MS eluents included sodium acetate (≥99.0%, Sigma) and formic acid (for LC-MS ≥99.0%, VWR). All samples (unless otherwise noted) were run immediately after collection.

An Ultimate 3000 Thermo HPLC was used for all kinetic analysis. For all 6PPD, IPPD, and 6PPD-Q methods, the column was set to 30°C. A 150 x 4 mm C18 gravity column with 3 µM particle size was used (Macherey-Nagel) or a 150 x 3 mm Eclipse XDB-C18 column with 5 µM particle size. The eluent lines were (A) ACN, (B) pH 4.5 acetate buffer 5mM + 10% ACN, and (C) MQ + 10% ACN.

- For 6PPD, an isocratic method of 70% A, 30% B was run at 1.15 mL/min for 4.3 minutes and absorbance was monitored at 290 nm (R.T. 3.7 min). The 6PPD-Q method was the exact same, but wavelength was monitored at 366 nm (R.T. 1.96 min).
- For IPPD, an isocratic method of 58% A, 42% B was run at 1.00 mL/min for 3.8 minutes and absorbance was monitored at 284 nm (R.T. 2.73 min).
- For PNA, an isocratic method of 50% A, 50% C was run at 1.00 mL/min for 5.0 minutes and absorbance was monitored at 316 nm (R.T. 2.76 min).

- For FFA, an isocratic method of 10% A, 90% C was run at 1.00 mL/min for 4.0 minutes and absorbance was monitored at 219 nm (R.T. 1.40 min).
- For $MNPO_2$ experiments a ramping method at 1.00 mL/min was run to elute both FFA and 6PPD. It began with 2% B, 98% C and ramped to 10% B, 90% C at time 1.5 min. It stayed stable until 1.95 min when it continued ramping to 80% B, 20% C until 4 min. It stayed stable here until 5 min where it switched immediately back to the starting conditions of 2% B, 98% C until the end of the run at 7.5 min.

To check for the presence of 6PPD-Q, a set of samples was run on a HPLC-QQQ MS (Vanquish HPLC + TSQ Quantis Plus MS, Thermo Scientific) with a C18 150 x 4.6 mm Ascentis Express column with 5 uM particle size (Supelco). The eluent lines were (A) MQ + 0.1% FA and (B) MeOH + 0.1% FA. The method had a 0.45 mL/min flow rate and proceeded as follows: from 0-5 min 30% A, 70% B, then from 5-6 min ramped to 5% A, 95% B where the method became isocratic until minute 12.5. From 12.5-12.7 min the method ramped back to 30% A, 70% B and maintained to re-equilibrate until the method ended at 15 min. The MS was run with an H-ESI source using selective reaction monitoring in positive mode. The positive ion voltage was 4700 V, sheath gas was 50 arb, aux gas 15 arb, sweep gas 0 arb. Ion transfer temp 350 °C and vaporizing temp 150 °C. The 6PPD and 6PPD-Q retention times were about 3.6 and 10.8 min, respectively.

Table S1: MS parameters for 6PPD and 6PPD-Q qualification and quantification

Compound of Interest	Precursor (m/z)	Product (m/z)	Collision Energy (V)
6PPD quantifier	269.201	183.967	20.61

6PPD qualifier	269.201	92.883	34.14
6PPD qualifier	269.201	106.967	47.61
6PPD-Q quantifier	299.2	240.967	29.28
6PPD-Q qualifier	299.2	186.967	27.99
6PPD-Q qualifier	299.2	214.967	16.53

S10. Electrochemistry of 6PPD and IPPD

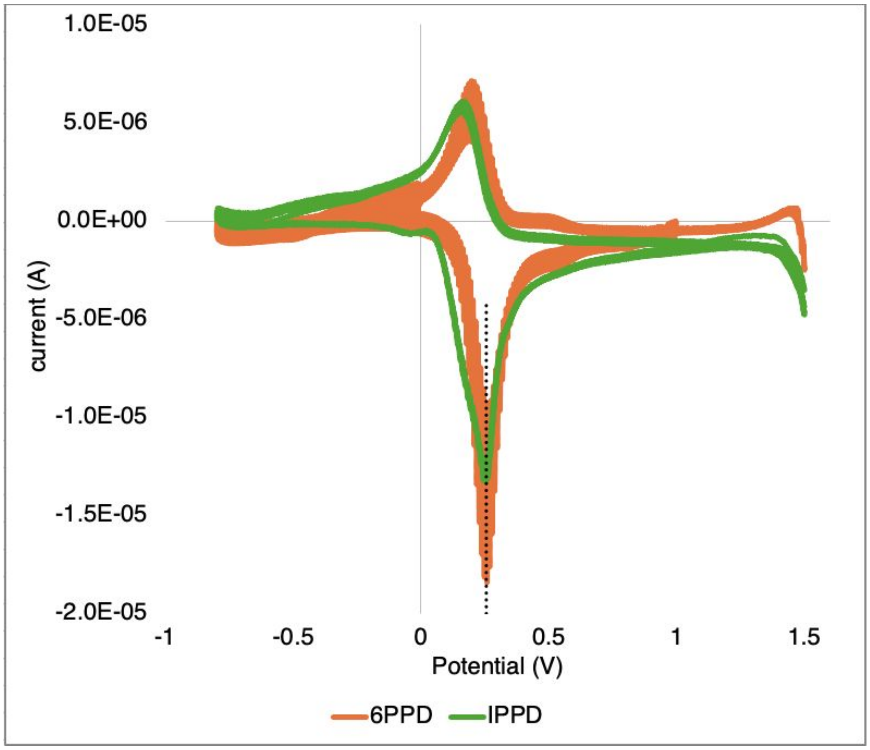

Figure S4: Cyclic voltammograms of 6PPD and IPPD. Electrochemistry performed in N_2 purged-water (pH 7) with sodium sulfate (0.5 M) as an electrolyte and a Ag/AgCl reference electrode. The black dotted vertical line marks the oxidation potential of the chemicals in these conditions, ~0.26 V.

S11. Ionic Strength Effects

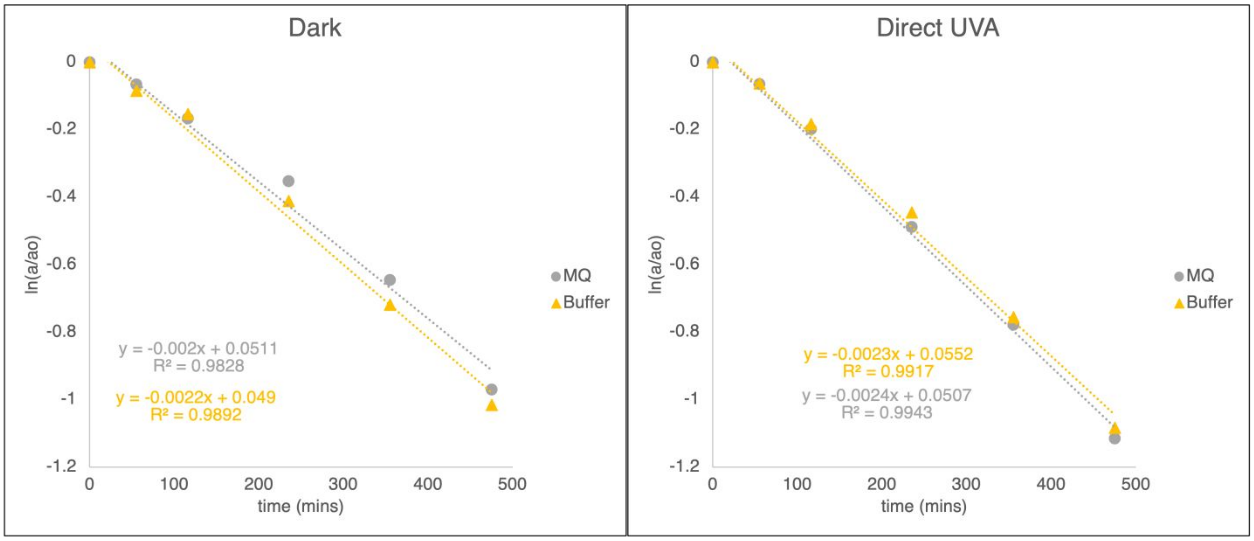

Figure S5: Testing the effects of ionic strength on 6PPD reaction rate. Test tubes were run side by side in the Rayonet photoreactor, but 6PPD was dissolved in either MQ or 12.5 mM pH 7 phosphate buffer.

S12. Molar Absorbance Spectra of 6PPD, IPPD, and 6-PPD-Q

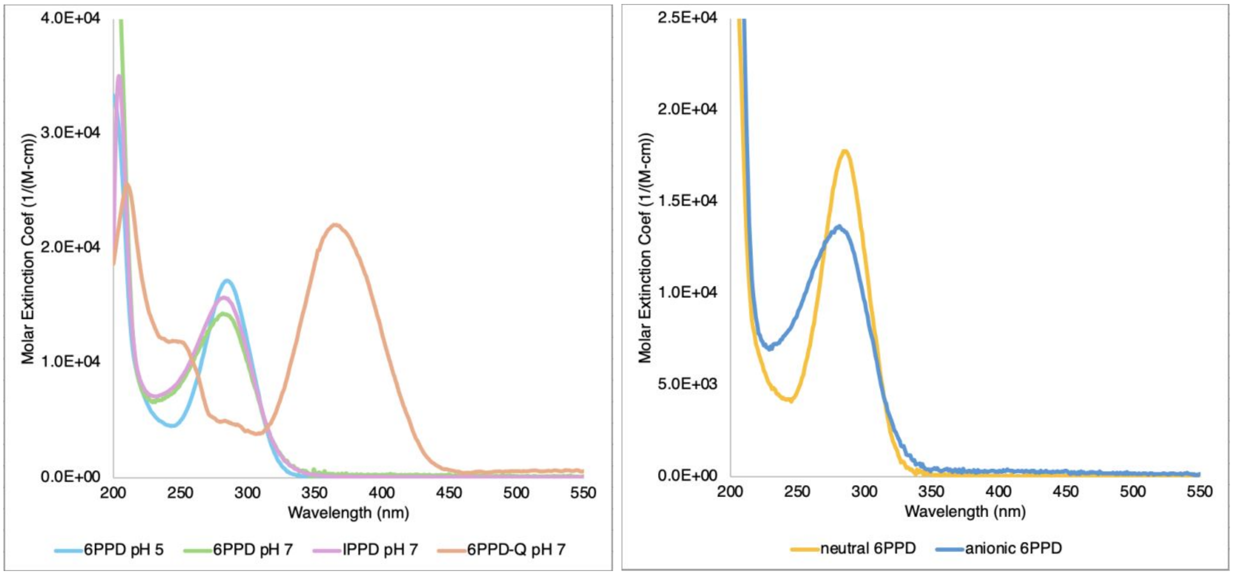

Figure S6: Left: Molar absorptivity of $6PPD_{pH\ 7}$, $6PPD_{pH\ 5}$, $IPPD_{pH\ 7}$, and $6PPD\text{-}Q_{pH\ 7}$.

Right: Molar absorptivity of the neutral and anionic forms of 6PPD.

Table S2: Molar extinction coefficients ($M^{-1}cm^{-1}$) for the neutral and anionic form of 6PPD and for 6PPD-Q as a function of wavelength (λ).

λ (nm)	*Neutral 6PPD*	*Anionic 6PPD*	*6PPD-Q*	*λ (nm)*	*Neutral 6PPD*	*Anionic 6PPD*	*6PPD-Q*	*λ (nm)*	*Neutral 6PPD*	*Anionic 6PPD*	*6PPD-Q*
550	8.29E+01	1.60E+02	5.95E+02	433	5.74E+01	2.57E+02	1.84E+03	316	3.63E+03	3.99E+03	4.66E+03
549	8.52E+01	1.47E+02	5.82E+02	432	4.13E+01	2.59E+02	2.00E+03	315	4.12E+03	4.12E+03	4.49E+03
548	-2.29E+01	7.27E+01	5.58E+02	431	9.45E+01	3.16E+02	2.15E+03	314	4.41E+03	4.37E+03	4.37E+03
547	-3.75E-02	1.21E+02	5.95E+02	430	-3.98E+01	2.33E+02	2.35E+03	313	5.01E+03	4.74E+03	4.23E+03
546	4.44E+01	8.51E+01	6.58E+02	429	8.59E+01	2.68E+02	2.56E+03	312	5.37E+03	5.03E+03	4.10E+03
545	2.04E+01	1.39E+02	6.79E+02	428	4.14E+01	2.71E+02	2.79E+03	311	5.95E+03	5.43E+03	3.94E+03
544	3.71E+00	1.11E+02	6.79E+02	427	4.48E+01	2.25E+02	3.03E+03	310	6.51E+03	5.79E+03	3.92E+03
543	3.30E+01	1.37E+02	6.28E+02	426	5.83E+01	3.43E+02	3.26E+03	309	7.16E+03	6.27E+03	3.86E+03
542	4.50E+01	1.41E+02	6.18E+02	425	5.64E+01	2.89E+02	3.44E+03	308	7.62E+03	6.56E+03	3.90E+03
541	6.77E+01	1.46E+02	6.02E+02	424	3.67E+01	3.30E+02	3.72E+03	307	8.04E+03	7.02E+03	3.84E+03
540	1.18E+02	2.18E+02	6.34E+02	423	1.03E+02	2.57E+02	4.01E+03	306	8.65E+03	7.41E+03	3.83E+03
539	5.16E+01	1.74E+02	6.73E+02	422	4.09E+01	2.77E+02	4.37E+03	305	9.13E+03	7.67E+03	3.85E+03
538	7.89E+01	1.86E+02	6.63E+02	421	9.34E+01	3.03E+02	4.70E+03	304	9.59E+03	7.97E+03	3.93E+03
537	2.98E+01	1.51E+02	6.77E+02	420	6.02E+01	3.09E+02	5.06E+03	303	1.03E+04	8.30E+03	3.94E+03
536	2.22E+01	1.18E+02	6.88E+02	419	7.37E+01	2.78E+02	5.34E+03	302	1.09E+04	8.81E+03	4.00E+03
535	8.71E+01	1.90E+02	6.30E+02	418	6.99E+00	2.59E+02	5.61E+03	301	1.15E+04	9.05E+03	4.05E+03
534	7.53E+01	1.58E+02	5.96E+02	417	4.73E+01	3.14E+02	5.91E+03	300	1.21E+04	9.53E+03	4.14E+03
533	3.88E+01	1.35E+02	5.83E+02	416	-4.17E+01	2.97E+02	6.24E+03	299	1.25E+04	9.73E+03	4.10E+03
532	3.20E+01	1.62E+02	5.98E+02	415	3.90E+01	2.76E+02	6.56E+03	298	1.33E+04	1.02E+04	4.07E+03
531	7.90E+01	1.47E+02	6.19E+02	414	3.42E+01	3.17E+02	6.92E+03	297	1.36E+04	1.05E+04	4.19E+03
530	6.66E+00	1.62E+02	6.50E+02	413	3.95E+01	2.79E+02	7.28E+03	296	1.43E+04	1.08E+04	4.32E+03
529	3.52E+01	1.69E+02	6.62E+02	412	-8.84E+00	2.92E+02	7.65E+03	295	1.48E+04	1.12E+04	4.46E+03
528	3.80E+01	1.45E+02	6.58E+02	411	4.85E+00	2.47E+02	8.07E+03	294	1.52E+04	1.15E+04	4.54E+03
527	7.90E+01	1.91E+02	6.50E+02	410	3.36E+01	3.18E+02	8.46E+03	293	1.58E+04	1.18E+04	4.61E+03
526	5.47E+01	1.05E+02	6.00E+02	409	9.36E+01	3.29E+02	8.83E+03	292	1.61E+04	1.22E+04	4.67E+03
525	3.43E+01	1.55E+02	6.05E+02	408	2.91E+01	3.25E+02	9.16E+03	291	1.66E+04	1.24E+04	4.62E+03
524	4.49E+01	1.29E+02	6.31E+02	407	4.99E+01	3.35E+02	9.60E+03	290	1.69E+04	1.27E+04	4.68E+03
523	2.90E+01	1.66E+02	6.33E+02	406	4.37E+01	4.18E+02	1.00E+04	289	1.73E+04	1.29E+04	4.70E+03
522	2.99E+01	1.67E+02	6.04E+02	405	8.34E+01	3.33E+02	1.04E+04	288	1.75E+04	1.30E+04	4.73E+03
521	2.39E+01	1.35E+02	5.96E+02	404	-1.03E+01	3.01E+02	1.08E+04	287	1.76E+04	1.32E+04	4.82E+03
520	1.77E+01	1.33E+02	5.80E+02	403	2.05E+01	3.50E+02	1.12E+04	286	1.78E+04	1.33E+04	4.82E+03
519	5.02E+01	1.31E+02	5.73E+02	402	-1.30E+01	2.50E+02	1.16E+04	285	1.77E+04	1.34E+04	4.91E+03

518	3.92E+01	1.44E+02	5.86E+02	401	1.56E+01	2.87E+02	1.21E+04	284	1.78E+04	1.35E+04	4.93E+03
517	3.21E+01	1.33E+02	5.77E+02	400	6.78E+01	3.29E+02	1.25E+04	283	1.75E+04	1.35E+04	5.00E+03
516	-1.77E+00	1.48E+02	6.13E+02	399	-2.55E+01	2.42E+02	1.30E+04	282	1.75E+04	1.36E+04	4.96E+03
515	2.28E+01	1.15E+02	6.28E+02	398	2.86E+01	2.88E+02	1.35E+04	281	1.73E+04	1.37E+04	4.94E+03
514	7.25E+01	1.70E+02	6.64E+02	397	4.00E+01	2.61E+02	1.39E+04	280	1.70E+04	1.36E+04	4.90E+03
513	4.71E+01	1.71E+02	6.41E+02	396	4.00E+01	3.26E+02	1.43E+04	279	1.67E+04	1.36E+04	4.89E+03
512	3.15E+01	1.76E+02	6.52E+02	395	6.42E+01	3.33E+02	1.47E+04	278	1.64E+04	1.35E+04	4.95E+03
511	-2.04E+00	1.08E+02	6.38E+02	394	3.45E+01	3.24E+02	1.51E+04	277	1.59E+04	1.33E+04	5.00E+03
510	5.67E+00	1.43E+02	6.36E+02	393	1.50E+01	2.88E+02	1.55E+04	276	1.57E+04	1.34E+04	5.13E+03
509	7.65E+01	1.69E+02	5.98E+02	392	7.62E+01	3.00E+02	1.59E+04	275	1.50E+04	1.34E+04	5.22E+03
508	5.52E+01	1.93E+02	5.96E+02	391	1.59E+01	2.41E+02	1.64E+04	274	1.45E+04	1.31E+04	5.27E+03
507	3.28E+01	1.72E+02	5.84E+02	390	1.08E+02	3.08E+02	1.68E+04	273	1.41E+04	1.31E+04	5.36E+03
506	5.21E+01	1.53E+02	5.83E+02	389	7.04E+01	3.33E+02	1.72E+04	272	1.36E+04	1.30E+04	5.41E+03
505	9.34E+00	1.56E+02	6.11E+02	388	7.12E+01	2.56E+02	1.76E+04	271	1.30E+04	1.28E+04	5.55E+03
504	5.02E+01	1.68E+02	5.74E+02	387	-1.72E+01	2.99E+02	1.78E+04	270	1.26E+04	1.28E+04	5.80E+03
503	-2.41E+01	1.56E+02	5.71E+02	386	7.95E+01	2.72E+02	1.82E+04	269	1.19E+04	1.24E+04	6.15E+03
502	3.78E+01	1.38E+02	6.01E+02	385	7.22E+01	2.68E+02	1.85E+04	268	1.15E+04	1.24E+04	6.58E+03
501	-1.21E+01	1.72E+02	5.88E+02	384	5.72E+01	3.64E+02	1.88E+04	267	1.09E+04	1.22E+04	6.95E+03
500	5.94E+01	1.78E+02	5.97E+02	383	4.45E+01	2.28E+02	1.91E+04	266	1.03E+04	1.20E+04	7.49E+03
499	-7.11E+00	1.33E+02	6.03E+02	382	2.97E+01	2.35E+02	1.93E+04	265	9.86E+03	1.19E+04	7.95E+03
498	7.89E+01	1.96E+02	5.88E+02	381	8.96E+01	3.68E+02	1.96E+04	264	9.38E+03	1.18E+04	8.43E+03
497	5.46E+01	1.94E+02	5.95E+02	380	1.09E+01	3.03E+02	1.99E+04	263	8.93E+03	1.15E+04	8.89E+03
496	5.92E+00	1.48E+02	6.08E+02	379	6.65E+01	2.70E+02	2.01E+04	262	8.37E+03	1.14E+04	9.25E+03
495	1.22E+02	2.37E+02	5.86E+02	378	9.78E+01	3.27E+02	2.04E+04	261	8.10E+03	1.12E+04	9.52E+03
494	-3.88E+00	1.62E+02	5.80E+02	377	1.44E+02	3.09E+02	2.07E+04	260	7.65E+03	1.09E+04	9.85E+03
493	2.23E+01	2.02E+02	5.62E+02	376	3.99E+01	2.70E+02	2.09E+04	259	7.20E+03	1.07E+04	1.01E+04
492	-2.31E+00	1.59E+02	5.70E+02	375	8.24E+01	2.95E+02	2.11E+04	258	6.93E+03	1.06E+04	1.04E+04
491	4.15E+01	1.87E+02	5.72E+02	374	1.03E+02	3.05E+02	2.12E+04	257	6.52E+03	1.03E+04	1.07E+04
490	2.34E+01	2.23E+02	5.31E+02	373	1.63E+02	4.45E+02	2.14E+04	256	6.26E+03	1.01E+04	1.11E+04
489	5.33E+01	1.38E+02	5.45E+02	372	1.25E+01	2.17E+02	2.15E+04	255	5.97E+03	9.95E+03	1.13E+04
488	1.34E+01	2.08E+02	5.61E+02	371	9.00E+00	2.50E+02	2.16E+04	254	5.55E+03	9.70E+03	1.15E+04
487	4.31E+00	1.55E+02	5.84E+02	370	1.85E+02	4.16E+02	2.17E+04	253	5.40E+03	9.53E+03	1.16E+04
486	-3.95E+00	1.63E+02	5.67E+02	369	7.47E+01	2.44E+02	2.18E+04	252	5.12E+03	9.30E+03	1.18E+04
485	1.58E+01	2.04E+02	5.69E+02	368	7.96E+01	3.06E+02	2.20E+04	251	4.92E+03	9.16E+03	1.18E+04
484	2.81E+01	1.78E+02	5.64E+02	367	1.15E+02	4.05E+02	2.20E+04	250	4.74E+03	9.01E+03	1.19E+04
483	3.79E+01	1.80E+02	5.54E+02	366	8.14E+01	3.18E+02	2.21E+04	249	4.57E+03	8.73E+03	1.19E+04

482	-7.93E+00	1.71E+02	5.30E+02	365	1.06E+02	2.61E+02	2.20E+04	248	4.41E+03	8.64E+03	1.19E+04
481	3.30E+01	1.63E+02	4.98E+02	364	9.72E+01	3.62E+02	2.21E+04	247	4.29E+03	8.54E+03	1.19E+04
480	-5.26E+00	1.80E+02	4.95E+02	363	1.59E+02	3.97E+02	2.20E+04	246	4.27E+03	8.37E+03	1.19E+04
479	-2.47E+01	1.73E+02	4.83E+02	362	1.17E+01	3.44E+02	2.19E+04	245	4.07E+03	8.18E+03	1.19E+04
478	-5.02E+01	1.76E+02	4.81E+02	361	5.52E+01	4.21E+02	2.18E+04	244	4.23E+03	8.07E+03	1.20E+04
477	-1.48E+01	1.77E+02	4.64E+02	360	1.35E+02	4.17E+02	2.17E+04	243	4.19E+03	7.98E+03	1.19E+04
476	4.97E+01	2.02E+02	4.83E+02	359	1.24E+02	3.61E+02	2.15E+04	242	4.25E+03	7.89E+03	1.19E+04
475	8.42E+01	2.88E+02	5.24E+02	358	5.55E+01	3.37E+02	2.13E+04	241	4.21E+03	7.81E+03	1.19E+04
474	-5.74E+01	1.58E+02	5.41E+02	357	1.00E+02	3.00E+02	2.11E+04	240	4.23E+03	7.69E+03	1.19E+04
473	4.76E+01	2.26E+02	5.64E+02	356	7.05E+01	3.39E+02	2.09E+04	239	4.32E+03	7.66E+03	1.20E+04
472	1.19E+01	2.26E+02	5.61E+02	355	1.00E+02	3.51E+02	2.06E+04	238	4.28E+03	7.50E+03	1.21E+04
471	-2.46E+01	2.26E+02	5.56E+02	354	8.15E+01	4.78E+02	2.02E+04	237	4.38E+03	7.48E+03	1.22E+04
470	2.74E+01	2.51E+02	5.42E+02	353	1.00E+02	4.00E+02	1.99E+04	236	4.41E+03	7.32E+03	1.22E+04
469	4.43E+01	2.11E+02	5.32E+02	352	8.92E+01	4.62E+02	1.98E+04	235	4.52E+03	7.28E+03	1.22E+04
468	4.04E+01	2.52E+02	5.07E+02	351	1.00E+02	4.00E+02	1.92E+04	234	4.51E+03	7.23E+03	1.23E+04
467	2.76E+01	1.81E+02	4.73E+02	350	1.08E+02	4.15E+02	1.88E+04	233	4.76E+03	7.24E+03	1.25E+04
466	3.69E+00	2.06E+02	4.22E+02	349	2.30E+02	3.00E+02	1.83E+04	232	4.86E+03	7.22E+03	1.28E+04
465	-7.95E+01	1.81E+02	4.56E+02	348	1.17E+02	4.00E+02	1.78E+04	231	4.78E+03	6.98E+03	1.30E+04
464	5.52E+00	1.98E+02	4.44E+02	347	1.63E+02	4.00E+02	1.73E+04	230	5.00E+03	7.11E+03	1.34E+04
463	4.42E+01	2.54E+02	4.63E+02	346	1.59E+02	4.00E+02	1.68E+04	229	5.06E+03	6.93E+03	1.36E+04
462	3.64E+01	2.08E+02	4.52E+02	345	3.81E+02	4.32E+02	1.63E+04	228	5.23E+03	7.04E+03	1.40E+04
461	3.35E+01	2.80E+02	4.37E+02	344	5.73E+01	4.96E+02	1.58E+04	227	5.43E+03	7.06E+03	1.43E+04
460	2.12E+01	1.62E+02	4.46E+02	343	2.81E+02	6.93E+02	1.53E+04	226	5.67E+03	7.22E+03	1.46E+04
459	2.06E+01	2.40E+02	4.23E+02	342	2.62E+02	5.53E+02	1.48E+04	225	5.78E+03	7.17E+03	1.52E+04
458	-5.57E+01	1.33E+02	4.98E+02	341	3.22E+02	7.17E+02	1.44E+04	224	6.10E+03	7.37E+03	1.57E+04
457	8.95E-01	1.95E+02	5.59E+02	340	2.04E+02	6.81E+02	1.39E+04	223	6.27E+03	7.45E+03	1.63E+04
456	1.35E+01	2.39E+02	5.52E+02	339	3.93E+02	6.93E+02	1.34E+04	222	6.53E+03	7.75E+03	1.69E+04
455	-2.47E+01	1.82E+02	5.61E+02	338	1.06E+02	6.46E+02	1.29E+04	221	6.77E+03	7.82E+03	1.75E+04
454	3.99E+01	2.57E+02	5.77E+02	337	2.33E+02	8.93E+02	1.23E+04	220	7.04E+03	8.32E+03	1.83E+04
453	-3.09E+01	1.57E+02	6.21E+02	336	2.87E+02	8.59E+02	1.18E+04	219	7.26E+03	8.67E+03	1.91E+04
452	4.54E+00	2.08E+02	6.50E+02	335	4.12E+02	9.55E+02	1.14E+04	218	7.77E+03	9.34E+03	2.00E+04
451	6.31E+01	2.82E+02	6.64E+02	334	3.77E+02	1.17E+03	1.09E+04	217	8.09E+03	9.91E+03	2.09E+04
450	7.88E+01	3.16E+02	6.91E+02	333	4.67E+02	1.15E+03	1.05E+04	216	8.54E+03	1.08E+04	2.20E+04
449	5.14E+01	3.41E+02	7.16E+02	332	5.00E+02	1.17E+03	1.01E+04	215	9.54E+03	1.22E+04	2.30E+04
448	2.76E+01	2.48E+02	7.66E+02	331	4.79E+02	1.21E+03	9.64E+03	214	1.05E+04	1.38E+04	2.39E+04
447	4.27E+01	2.95E+02	7.69E+02	330	6.39E+02	1.45E+03	9.09E+03	213	1.17E+04	1.64E+04	2.47E+04

446	-3.95E+01	2.69E+02	7.58E+02	329	7.90E+02	1.66E+03	8.65E+03	212	1.31E+04	1.91E+04	2.52E+04
445	3.52E+01	2.67E+02	8.08E+02	328	8.47E+02	1.65E+03	8.25E+03	211	1.51E+04	2.23E+04	2.55E+04
444	1.26E+02	2.94E+02	8.44E+02	327	7.50E+02	1.67E+03	7.78E+03	210	1.72E+04	2.56E+04	2.56E+04
443	4.22E+01	2.74E+02	9.33E+02	326	1.03E+03	1.97E+03	7.51E+03	209	1.96E+04	2.94E+04	2.54E+04
442	2.86E+01	2.67E+02	9.85E+02	325	1.17E+03	2.08E+03	7.18E+03	208	2.20E+04	3.36E+04	2.48E+04
441	3.87E+01	2.58E+02	1.05E+03	324	1.44E+03	2.27E+03	6.78E+03	207	2.40E+04	3.78E+04	2.42E+04
440	8.77E+01	2.86E+02	1.11E+03	323	1.58E+03	2.44E+03	6.49E+03	206	2.59E+04	4.15E+04	2.35E+04
439	2.74E+01	2.03E+02	1.21E+03	322	1.77E+03	2.50E+03	6.15E+03	205	2.77E+04	4.56E+04	2.27E+04
438	3.67E+01	2.47E+02	1.29E+03	321	1.95E+03	2.83E+03	5.79E+03	204	2.91E+04	5.00E+04	2.19E+04
437	4.03E+01	2.38E+02	1.35E+03	320	2.23E+03	3.02E+03	5.51E+03	203	3.03E+04	5.52E+04	2.12E+04
436	1.40E+01	2.49E+02	1.46E+03	319	2.44E+03	3.23E+03	5.26E+03	202	3.12E+04	6.02E+04	2.03E+04
435	9.80E+01	3.11E+02	1.59E+03	318	2.87E+03	3.43E+03	5.02E+03	201	3.19E+04	6.62E+04	1.95E+04
434	5.11E+01	2.79E+02	1.72E+03	317	3.24E+03	3.59E+03	4.75E+03	200	3.29E+04	7.39E+04	1.87E+04

S13. UVA vs UVB Direct Photochemistry Kinetic Data

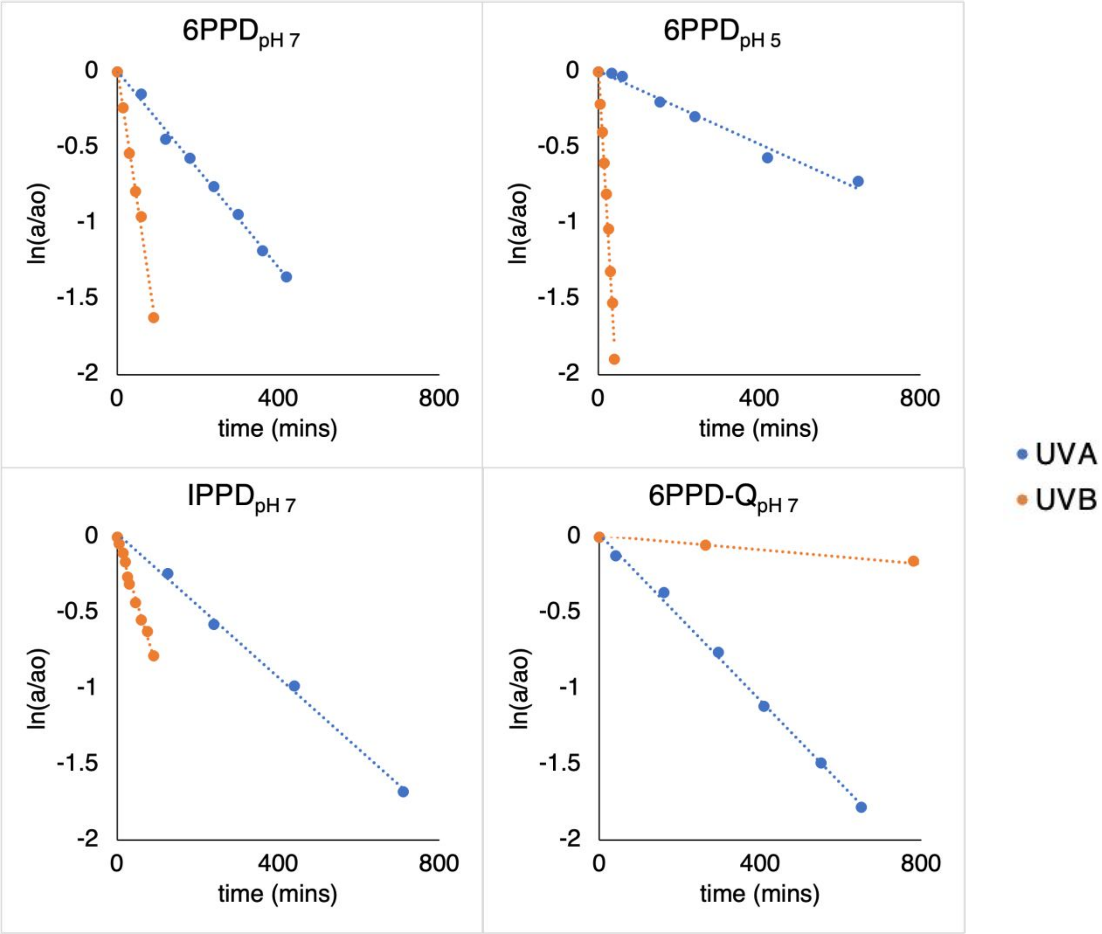

Figure S7: The kinetics of irradiation experiments in UVA and UVB light are shown for each chemical $6PPD_{pH\ 7}$, $6PPD_{pH\ 5}$, $IPPD_{pH\ 7}$, and $6PPD\text{-}Q_{pH\ 7}$.

S14. Dark and Direct UVA Photochemistry

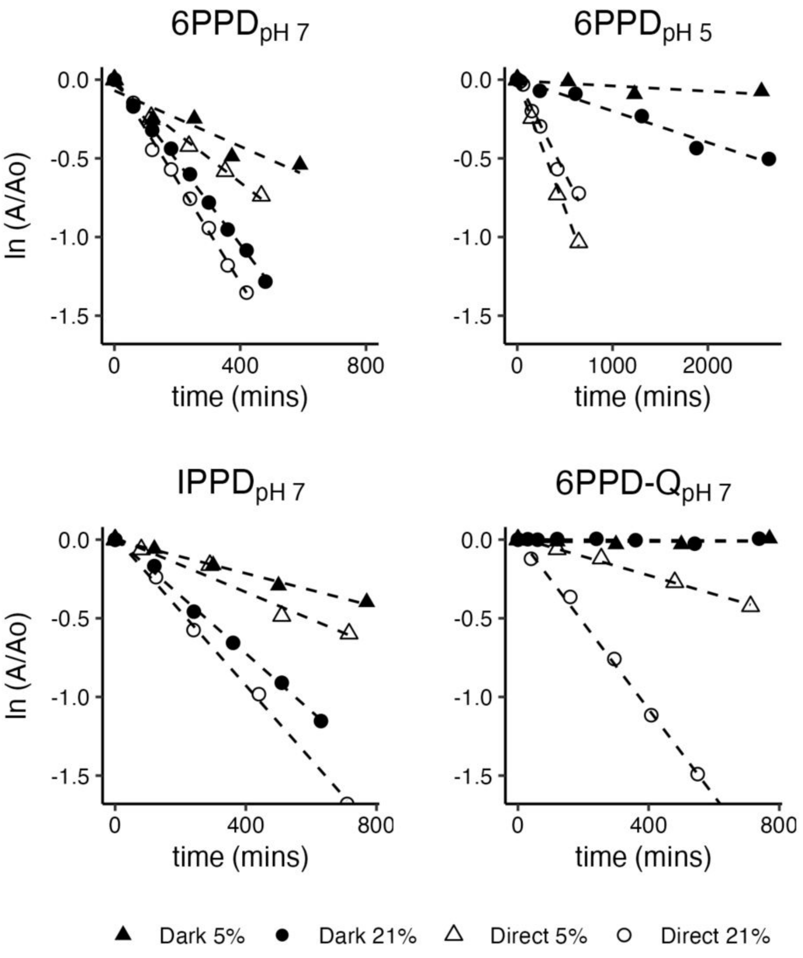

Figure S8: Dark and direct photochemical degradation of 6PPD$_{pH\ 7}$, 6PPD$_{pH\ 5}$, IPPD$_{pH\ 7}$, and 6PPD-Q$_{pH\ 7}$ in 21% and 5% oxygen conditions.

S15. SRNOM Steady-State Kinetic Data

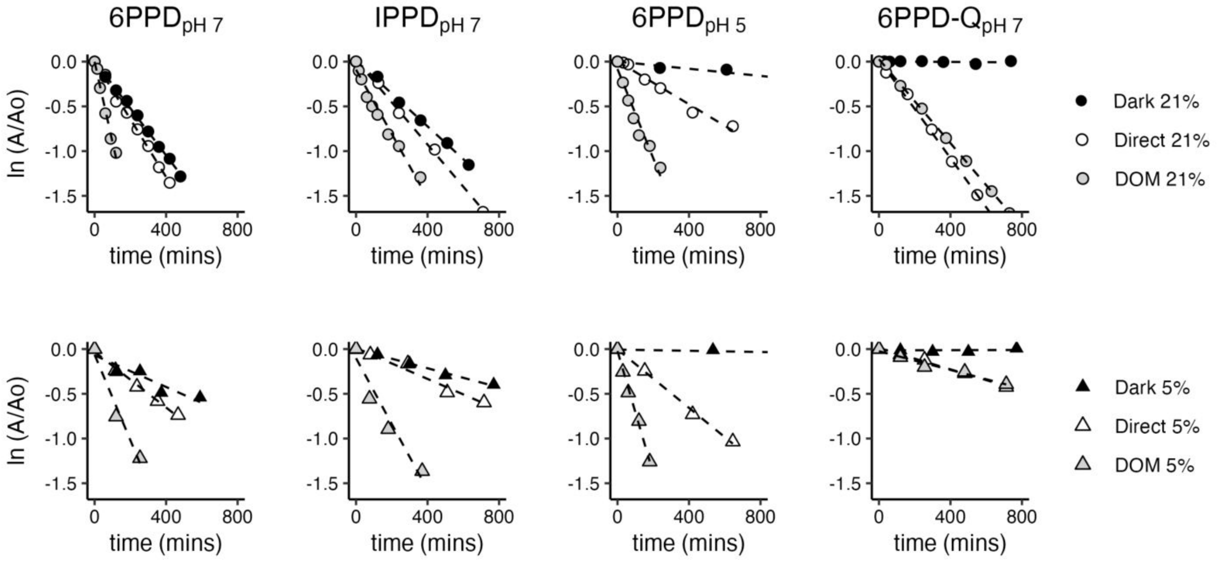

Figure S9: Reaction of $6PPD_{pH\ 7}$, $IPPD_{pH\ 7,}$ $6PPD_{pH\ 5}$, and $6PPD\text{-}Q_{pH\ 7}$, with SRNOM at 21% O_2 and 5% O_2 conditions. The dark and direct data is the same as in Figure 2 in the main paper, but the DOM data is different as here SRNOM was used rather than MRNOM.

S16. Steady-State $^1O_2$

The $^1O_2$ bimolecular reaction rate constants (k^{Δ}_{tot}) are also shown in Table 3 of the main paper. These time-resolved values are a combination of physical and chemical quenching. For the reaction of 6PPD or IPPD with $^1O_2$, we believe that chemical quenching is minimal (as discussed below) and k^{Δ}_{tot} is approximately equal to the physical quenching rate constant. Again $6PPD_{pH\ 7}$ has the highest reactivity with $^1O_2$, compared to $6PPD_{pH\ 5}$ and $IPPD_{pH\ 7}$, although the values are all quite similar. As the quenching rate constants shown here include both physical and chemical quenching, testing steady-state reactivity is also important.

Unfortunately, the reactivity of 6PPD and IPPD with $^1O_2$ in steady-state conditions proved difficult to study. The low oxidation potentials of these PPDs makes them reactive with many of the porphyrin sensitizers typically used as well-defined sources of $^1O_2$. Although increased reactivity was seen in the presence of ZnP-produced $^1O_2$ for 6PPD and IPPD, the reaction rates were extremely similar in pure buffer and 50% D_2O conditions. Adding 50% D_2O should approximately double the concentration of $^1O_2$ in solution, due to a lower solvent relaxation rate. If the observed reactivity was due solely to $^1O_2$, the reaction rate would double, however we saw no significant increase in reaction rate (Figure S10). This suggests that increased reactivity due to the addition of the porphyrin was mostly due to reaction with the porphyrin itself, and not due to the production of $^1O_2$.

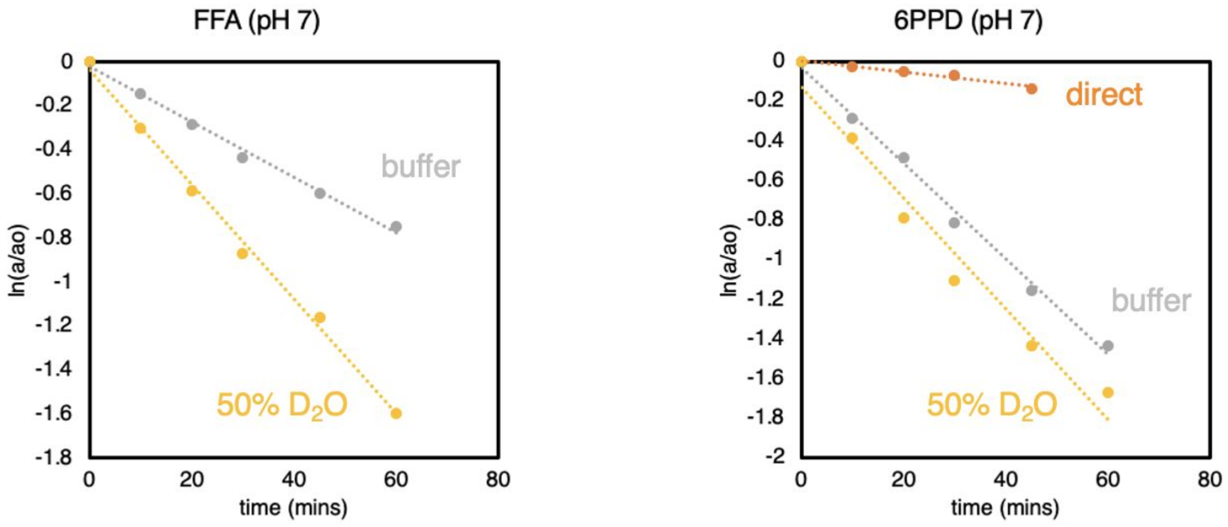

Figure S10: Reactivity of FFA and $6PPD_{pH\ 7}$ in 100% buffer or 50% D_2O / 50% buffer conditions, with the presence of $^1O_2$ production from ZnP. The direct degradation of 6PPD is also shown for comparison.

In transient absorption experiments with 6PPD and ZnP, evidence of $ZnP^{\bullet-}$ (signal around 725 nm) and a $6PPD^{\bullet+}$ (signal around 600 nm)[6,7] was observed, suggesting a direct electron transfer from 6PPD to ZnP (Figure S11).

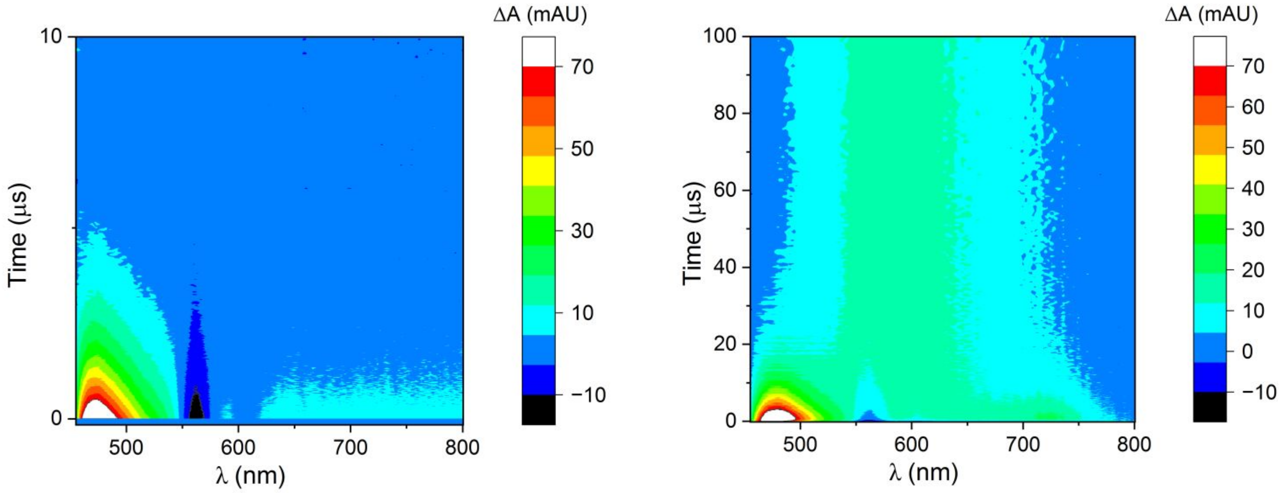

Figure S11: 3D transient absorption spectra of ZnP without (left) and with (right) addition of 1000 μM 6PPD upon 440 nm excitation, measured up to 10 μs and 100 μs, respectively. On the right, the new $6PPD^{\bullet+}$ (600 nm) and $ZnP^{\bullet-}$ (725 nm) signals are evidence of an electron transfer reaction.

The same effect occurred when using the endoperoxide, $MNPO_2$. We saw great reactivity in pure D_2O, but when the D_2O content was changed from 100% to 50%, no change in reactivity was seen (while it decreases as expected for FFA) (Figure S12). We hypothesize that 6PPD is also capable of being oxidized directly by $MNPO_2$. All we can conclude is that if there is chemical reactivity between PPD and $^1O_2$, it is very minimal and does not play a large role in the overall degradation of the compound. Other ways to produce $^1O_2$ cleanly are required to obtain a precise reaction rate constant.

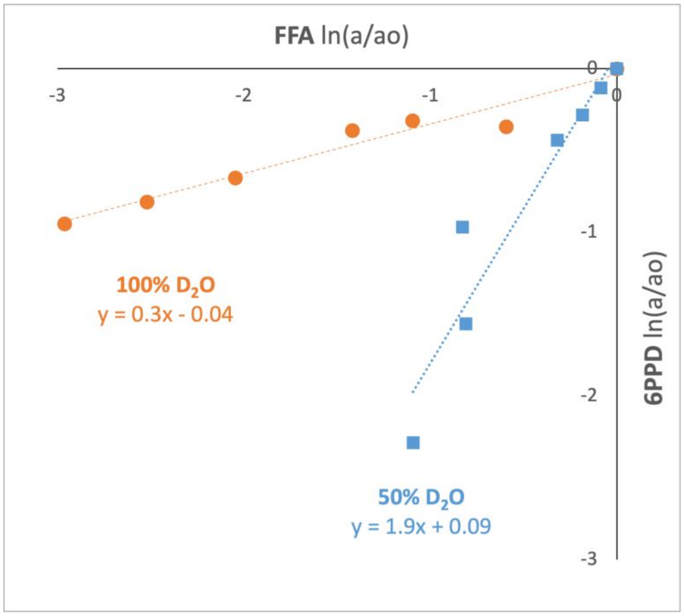

Figure S12: The relative reactivity of FFA and $6PPD_{pH\ 7}$ in 100% D_2O or 50% D_2O / 50% buffer conditions, with the presence of $^1O_2$ production from $MNPO_2$. $^1O_2$ should be roughly 7 times higher in 100% D_2O conditions and it seems that FFA degradation increased greatly while 6PPD did not.

For 6PPD-Q, the addition of ZnP-produced $^1O_2$ causes decreased degradation (
Figure S13). This suggests that only light screening from ZnP was observed and $^1O_2$ degradation is not expected to be a significant pathway either. Overall, these results suggest that direct photodegradation is by far the major pathway for 6PPD-Q, while dark and indirect reactions play a minimal role. This agrees with Redman et al, although they also noted that at lower temperatures (4°C and 12°C), indirect degradation plays an increasingly important role. The processes occurring during indirect photochemistry at lower temperatures is something that needs to be further studied.

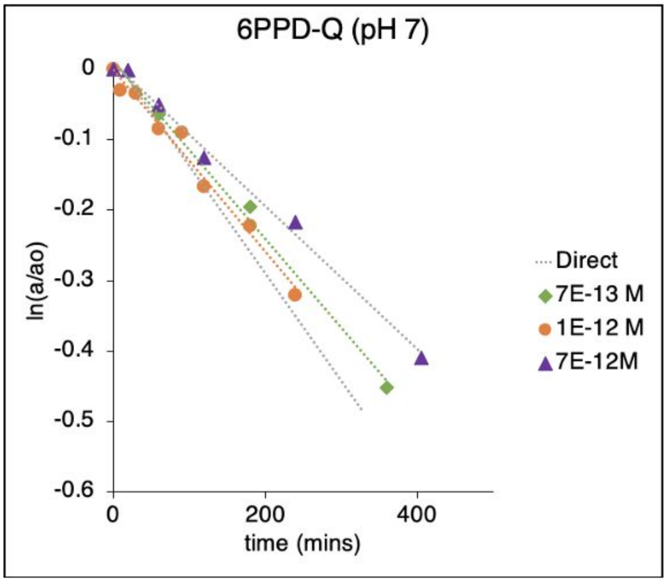

Figure S13: Degradation of 6PPD-Q via direct photochemistry vs. with the addition of 7E-13 M, 1E-12 M, or 7E-12 M $^1O_2$. Singlet oxygen produced via ZnP sensitization does not increase the degradation rate, but rather only screen light.

A recent study suggested that 6PPD could self-sensitize and produce $^1O_2$ on its own. In their study, Li *et al.* confirmed this with the observation of a $^1O_2$-adduct in their 6PPD system using electron paramagnetic resonance spectrometry.[8] They also noted that the energy differences between the excited state and ground state of 6PPD are all greater than the energy required to promote ground state oxygen to the singlet state.[8] We further tested this hypothesis by performing time-resolved $^1O_2$ phosphorescence measurements. We observed a 6PPD concentration-dependent production of $^1O_2$. At higher 6PPD concentrations, no $^1O_2$ phosphorescence was observed, while at lower concentrations, we observed a clear growth and decay of the $^1O_2$ phosphorescence signal. This suggests that 6PPD can both sensitize oxygen to create $^1O_2$ and then react with the $^1O_2$ produced. The quantum yield of this reaction was about 1%, which is quite low, especially in comparison to model sensitizers, such as perinaphthenone

which has a quantum yield of 0.98.[9] This result confirms that the $^1O_2$ pathway is of minimal importance.

S17. $^1O_2$ Phosphorescence Model Fits

SRNOM + 6PPD pH 7

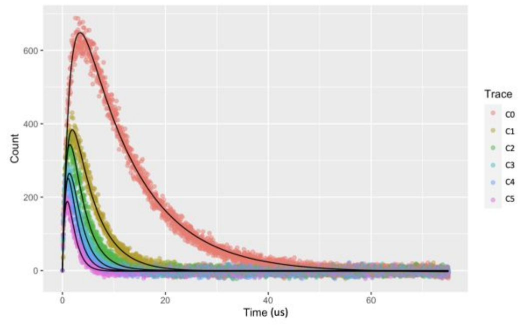

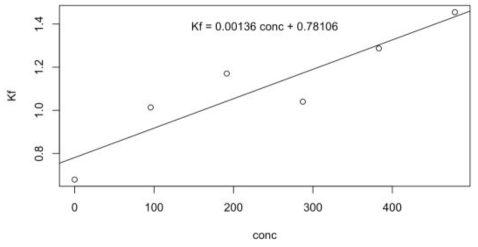

MRNOM + 6PPD pH 5

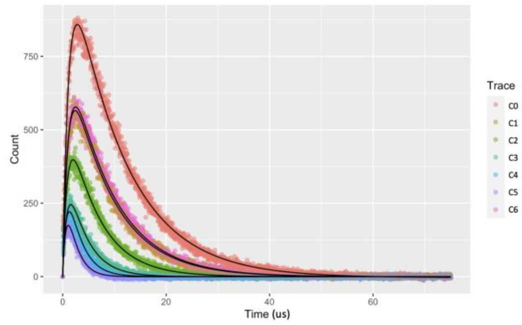

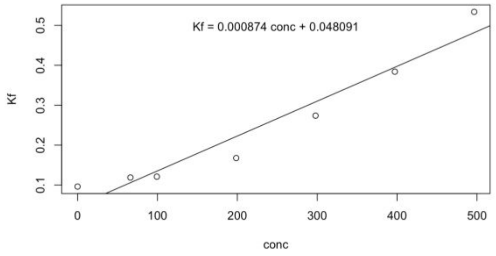

SRNOM + 6PPD pH 5

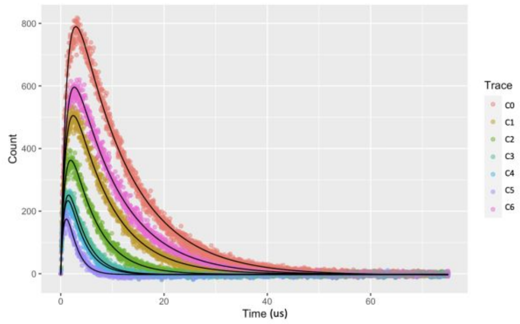

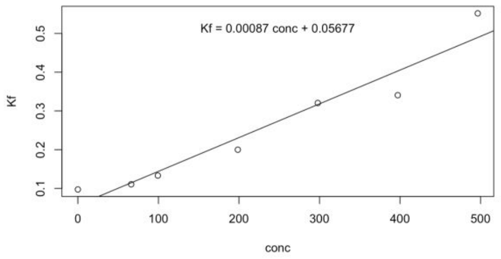

SRNOM + IPPD pH 7

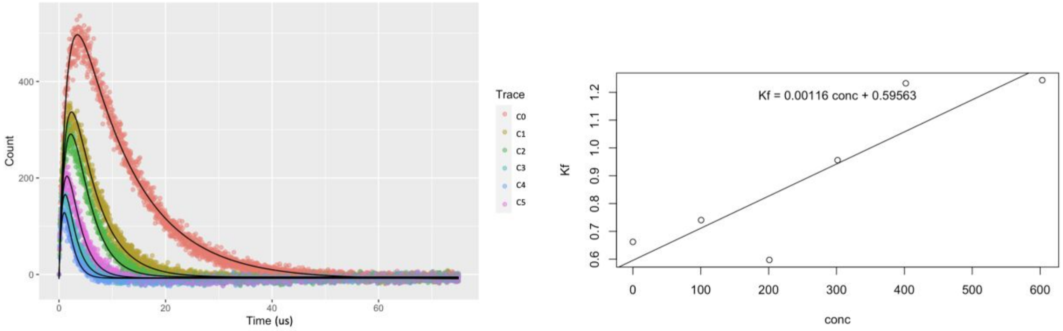

MRNOM + IPPD pH 7

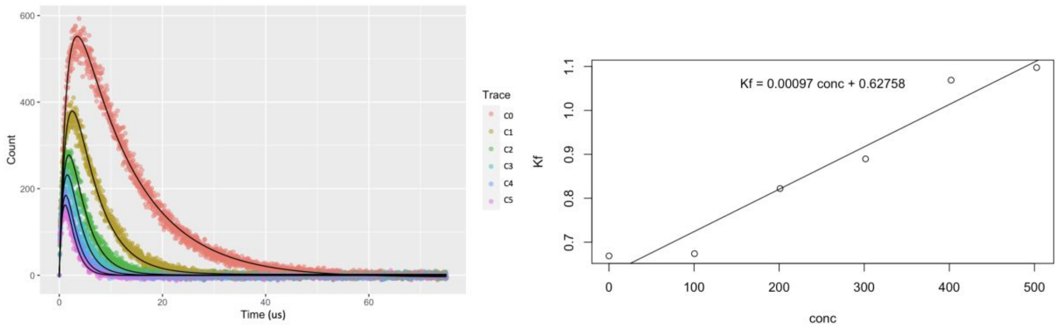

Figure S14: Singlet oxygen phosphorescence growth and decay curves (data with model fit) for DOM experiments. The $^1O_2$ signal is quenched as more 6PPD or IPPD is added. The slope of the Stern-Volmer plot of the growth rate vs. the PPD concentration (μM) provides the DOM triplet quenching rate constant for each PPD.

S18. Transient Absorption DOM + 6PPD

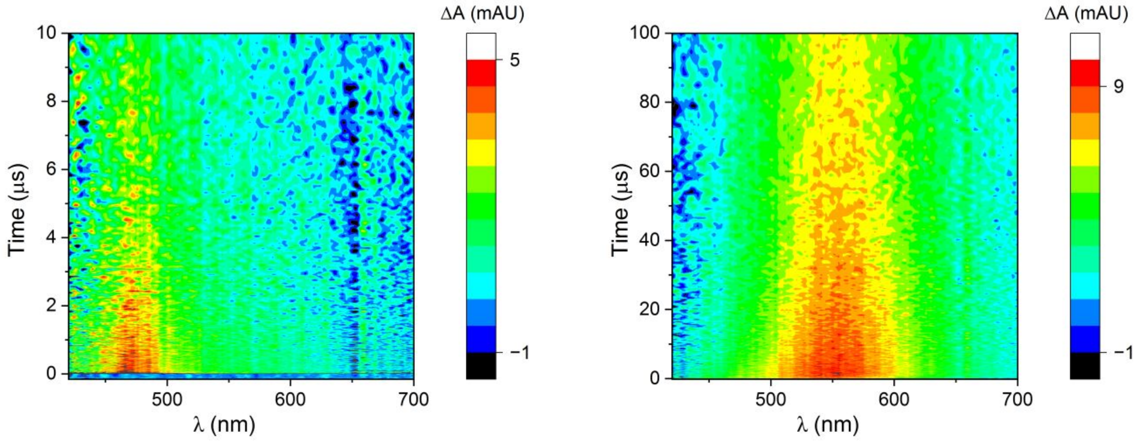

Figure S15: Example 3D transient absorption spectra of MRNOM without (left) and with (right) the addition of 1220 µM 6PPD (right) upon 365 nm excitation, presented up to 10 µs and 100 µs, respectively. The signal at 475 nm, assumed to form from the MRNOM, is shown on the left. With the addition of 6PPD, the 550 nm signal forms, which is assumed to be the 6PPD radical cation.

S19. 6PPD-Q Formation and Ozone Experiments

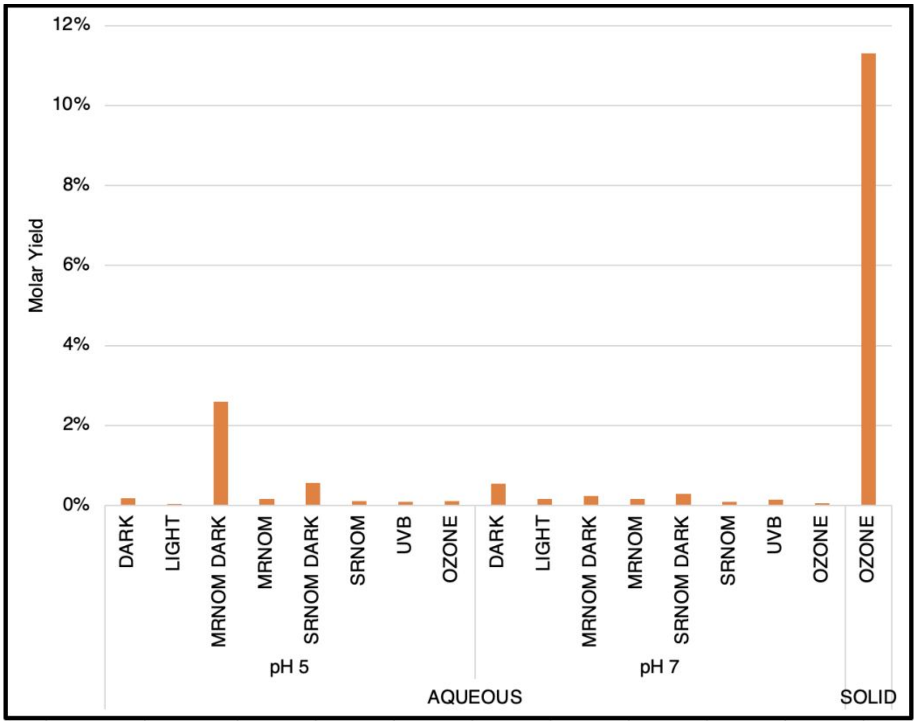

Figure S17: Molar yields for the production of 6PPD-Q from 6PPD from all tested aquatic degradation pathways, as well as from the ozonation of 6PPD in the solid-state on glass slides.

References

(1) Pierlot, C.; Aubry, J. M.; Briviba, K.; Sies, H.; Di Mascio, P. Naphthalene Endoperoxides as Generators of Singlet Oxygen in Biological Media. *Methods Enzymol* **2000**, *319*, 3–20. https://doi.org/10.1016/s0076-6879(00)19003-2.

(2) Nieuwint, A. W. M.; Aubry, J. M.; Arwert, F.; Kortbeek, H.; Herzberg, S.; Joenje, H. Inability of Chemically Generated Singlet Oxygen to Break the DNA Backbone. **2009**. https://doi.org/10.3109/10715768509056532.

(3) Fields, D. L. J.; Lodaya, J. S. Preparation of Quinoneimines from Hydroxyphenylamines Using a Hypochlorite as an Oxidation Agent. WO 99/52860, April 6, 1999.

(4) Team, R. C. R: A Language and Environment for Statistical Computing. R Foundation for Statistical Computing: Vienna, Austria 2016. http://www.r-project.org.

(5) Erickson, P. R.; Moor, K. J.; Werner, J. J.; Latch, D. E.; Arnold, W. A.; McNeill, K. Singlet Oxygen Phosphorescence as a Probe for Triplet-State Dissolved Organic Matter

Reactivity. *Environ Sci Technol* **2018**, *52* (16), 9170–9178. https://doi.org/10.1021/acs.est.8b02379.
(6) Rapta, P.; Vargová, A.; Polovková, J.; Gatial, A.; Omelka, L.; Majzlík, P.; Breza, M. A Variety of Oxidation Products of Antioxidants Based on N,N′-Substituted p-Phenylenediamines. *Polym Degrad Stab* **2009**, *94* (9), 1457–1466. https://doi.org/10.1016/J.POLYMDEGRADSTAB.2009.05.003.
(7) Zhou, Y.; Yixi, L.; Kong, Q.; Peng, J.; Pan, Y.; Qiu, J.; Yang, X. Sunlight-Induced Transformation of Tire Rubber Antioxidant N-(1,3-Dimethylbutyl)-N′-Phenyl-p-Phenylenediamine (6PPD) to 6PPD-Quinone in Water. *Cite This: Environ. Sci. Technol. Lett* **2023**, *10*, 803. https://doi.org/10.1021/acs.estlett.3c00499.
(8) Li, C.; Zhang, Y.; Yin, S.; Wang, Q.; Li, Y.; Liu, Q.; Liu, L.; Luo, X.; Chen, L.; Zheng, H.; Li, F. First Insights into 6PPD-Quinone Formation from 6PPD Photodegradation in Water Environment. *J Hazard Mater* **2023**, *459*, 304–3894. https://doi.org/10.1016/j.jhazmat.2023.132127.
(9) Schmidt, R.; Tanielian, C.; Dunsbach, R.; Wolff, C. Phenalenone, a Universal Reference Compound for the Determination of Quantum Yields of Singlet Oxygen O2(1Δg) Sensitization. *J Photochem Photobiol A Chem* **1994**, *79* (1–2), 11–17. https://doi.org/10.1016/1010-6030(93)03746-4.
